# Transcriptomic characterization of key psoriasis-associated genes based on single-cell RNA-seq and machine learning

**DOI:** 10.1371/journal.pone.0352663

**Published:** 2026-07-13

**Authors:** Weixiang Wang, Qiang Zhang, Suting Xu

**Affiliations:** Department of Clinical laboratory, The First Affiliated Hospital of Lishui University, Lishui People's Hospital, Lishui, Zhejiang, China; Shanghai Jiao Tong University, CHINA

## Abstract

**Background:**

Psoriasis is a multifaceted skin and systemic disorder driven by a complex interplay of genetic, immunological, and environmental factors. Genetic predisposition plays a pivotal role, with the IL-17/IL-23 immune axis recognized as a central pathogenic pathway. Ongoing research, however, continues to uncover additional critical drivers, cytokines, intracellular signaling networks, and potential therapeutic targets.

**Methods:**

Single-cell RNA sequencing (scRNA-seq) datasets comprising both psoriatic and healthy samples were obtained from the Gene Expression Omnibus (GEO). Cell-type proportions were estimated using Cell-type Identification by Estimating Relative Subsets of RNA Transcripts (CIBERSORT), and weighted gene co-expression network analysis (WGCNA) was applied to explore correlations between cell types and gene signatures. Machine learning algorithms were subsequently employed to identify four psoriasis-associated key genes: *DEFB4A*, *GJB2*, *SERPINB3*, and *SERPINB13*. Their expression was validated in bulk RNA-seq datasets. Using scRNA-seq data, we further investigated the lesional regulatory roles of these genes and their associated pathway alterations, and we proposed targeted therapeutic strategies.

**Results:**

A series of algorithms identified 271 hub genes significantly associated with psoriasis lesions and basal cells. Machine learning analysis refined this set to four key genes in psoriasis: *DEFB4A*, *GJB2*, *SERPINB3,* and *SERPINB13*.

**Conclusions:**

These four psoriasis-associated driver genes were upregulated in lesional skin. We also screened small-molecule compounds targeting these genes, offering potential therapeutic strategies.

## Introduction

Psoriasis is a chronic, recurrent systemic inflammatory disorder driven by environmental triggers, genetic predisposition, and multifaceted immune dysregulation [1–4]. It presents as scaly erythematous plaques that may be localized or widespread, affecting individuals of all ages without sex predilection [1–3]. In the psoriatic epidermis, basal keratinocytes constitute the main proliferative compartment and are central to the hyperproliferation and aberrant differentiation that characterize the disease [2]. Symptoms commonly fluctuate with the seasons, worsening in winter and improving in summer. The etiology is complex, and heredity plays a substantial role, with roughly 30% of patients reporting a family history [5–7]. Although the IL-17 and IL-23 pathways are recognized as key drivers, ongoing research continues to uncover additional genetic factors, cytokines, and intracellular signaling cascades that are critical for effective long-term management [2,3]. As psoriasis remains incurable, selecting the most appropriate individualized treatment is essential to control the disease, prevent debilitating complications, reduce disabling comorbidities, maintain therapeutic efficacy, and enhance overall quality of life [2,3].

Conventional RNA-seq is performed on bulk samples and captures the averaged genetic information of many cells [8]. In contrast, single-cell RNA sequencing (scRNA-seq) is a state-of-the-art high-throughput technique that provides gene expression profiles at single-cell resolution, revealing previously hidden cellular heterogeneity and enabling the investigation of cell-cell interactions [8,9]. Machine learning, encompassing predictive modeling and data,driven pattern recognition, has been applied in biology for decades and is now pervasive across the biological sciences [10].

In this study, we used scRNA-seq data from the Gene Expression Omnibus (GEO) to analyze cell population differences between psoriatic lesions and normal controls. Bulk RNA-seq datasets were also obtained from the same source. The CIBERSORT algorithm was applied for deconvolution analysis to quantify the abundance of distinct cell types, and weighted gene co-expression network analysis (WGCNA) was employed to identify co-regulated cell types and gene signatures in psoriasis. Machine learning techniques were then used to pinpoint four key genes, which were subsequently validated in the bulk RNA-seq data. Finally, we explored how these four genes regulate psoriatic lesions through altered pathway activity and proposed targeted therapeutic strategies based on the scRNA-seq findings.

## Materials and methods

### Data sources

Single-cell RNA-seq datasets comprising psoriasis and healthy samples were retrieved from the GEO database: GSE194315 (n = 24), GSE151177 (n = 23), GSE221648 (n = 10), and GSE162183 (n = 6). Bulk RNA-seq datasets for psoriasis and healthy samples were also obtained from GEO, including GSE186063 (n = 26), GSE54456 (n = 174), GSE121212 (n = 93), GSE66511 (n = 36), GSE117405 (n = 28), GSE205748 (n = 27), GSE114286 (n = 27), GSE83645 (n = 25), GSE142582 (n = 15), GSE41745 (n = 6), and GSE186117 (n = 5).

### Single-cell RNA-seq cohort analysis process

The Seurat package (v4.4.0) was used as the primary analysis tool for quality control (QC) of each of the four datasets. QC filtering was performed as follows: genes detected in fewer than 10 cells were removed; cells with fewer than 300 detected genes were discarded; and cells with mitochondrial gene percentage (percent.mt) exceeding 10% or hemoglobin gene percentage (percent.hb) exceeding 1% were excluded. The mitochondrial and hemoglobin gene percentages were calculated using the PercentageFeatureSet function with the patterns ^MT- and ^HB[^(p)], respectively. These thresholds were chosen based on the metric distributions observed in our dataset and are consistent with, or more stringent than, widely adopted defaults in scRNA-seq analysis.

The four datasets were merged, and Harmony (v1.0.3) was applied to correct for batch effects. Uniform Manifold Approximation and Projection (UMAP) dimensionality reduction was then run using the top 25 principal components (PCs) as input. For cell clustering, different resolution parameters were adopted for different cell subsets: 0.4 for all cells, 0.5 for lymphoid-lineage immune cells, and 0.6 for myeloid-lineage immune cells. Semi-supervised cell-type annotation was performed with SingleR (v2.0.0) in conjunction with the CellMarker database. The complete list of defining markers is provided in Supplementary1 Table. To obtain a broad representation of cellular features, all downstream analyses were performed on the globally integrated dataset without stratification by sample source (tissue versus peripheral blood).

### Integration of Bulk RNA-seq data

The 11 bulk RNA-seq cohorts were integrated by intersecting their gene sets, and batch effects were corrected using the removeBatchEffect function from the limma package (v3.54.2). Principal component analysis (PCA) was subsequently applied to confirm the effective removal of batch effects.

### Deconvolution of Bulk RNA-seq data using scRNA-seq reference signatures

Cell-type proportions in the bulk RNA-seq data were estimated with CIBERSORT. Reference signature matrices were built separately for healthy and psoriasis conditions using the single-cell data. For each condition, differential expression analysis (Seurat FindAllMarkers, only.pos = TRUE, min.pct = 0.5, logfc.threshold = 0.5) was performed to identify the top 50 upregulated genes for each cell subpopulation relative to all other cells. The average expression of these genes in each cell type was calculated and used as the reference matrix for deconvolution of the corresponding bulk transcriptomic cohort (healthy signature for healthy samples, lesional signature for psoriasis samples). This condition-specific design was chosen to capture potential disease-related transcriptional alterations; its limitations are addressed below.

### WGCNA identifies cell subpopulations highly associated with psoriatic lesions

Lesion and non-lesion samples were subject to WGCNA. After filtering lowly expressed genes and outlier samples, gene features were clustered into distinct co-expression modules. Clinical variables and CIBERSORT-estimated cell-type abundances were used as traits in subsequent correlation analyses to assess module-trait relationships. Hub genes were then extracted from the relevant modules using the following criteria: module membership (MM) > 0.8 and gene significance (GS) > 0.2.

### Machine learning dimensionality reduction of highly relevant gene features for psoriasis

To identify genes specifically upregulated in basal cells, differential expression analysis was performed comparing basal cells with all other cell types using the Seurat FindMarkers function (only.pos = TRUE, min.pct = 0.1, logfc.threshold = 0.25, adjusted P < 0.05). The resulting basal cell differential gene set was then intersected with the WGCNA hub genes. The overlapping genes, considered robust candidates associated with basal cell biology, were used as features for subsequent machine learning.

Machine learning classification was implemented using Scikit-learn in Python, employing a Linear Support Vector Machine (Linear SVM) and a Random Forest Classifier (RFC). The 74 candidate features were pre-selected from the intersection of WGCNA hub genes and basal cell differentially expressed genes using the full dataset; this pre-selection step was not independent of the validation data and is acknowledged as a limitation. Following pre-selection, the data were split into training and validation sets. Hyperparameter tuning via grid search and model calibration with 10-fold cross-validation were performed strictly within the training set. Feature importance scores were extracted from the trained models, and the top 30 features from each classifier were intersected to define the final key genes. Model performance was evaluated on the held-out validation set using the Area Under the Receiver Operating Characteristic Curve (AUC), which ranges from 0 to 1, with values closer to 1 indicating superior classification performance.

To enhance the precision of feature selection, two classification cohorts were constructed: psoriasis lesion versus non-lesion, and psoriasis lesion versus healthy control. For each task, the data were randomly partitioned into training and validation sets using Scikit-learn*'*s train_test_split function with a fixed random seed to ensure reproducibility.

### Exploring the relationship between key psoriasis-associated genes and basal cell differentiation

Pseudotime trajectory analysis of basal cells from psoriasis samples was performed using the Monocle package (v2.26.0). Highly variable genes selected by the Monocle algorithm were used to construct the pseudotime differentiation axis, and DDRTree was applied for nonlinear dimensionality reduction. The expression dynamics of key psoriasis-associated genes along the pseudotime trajectory were visualized as a heat map.

### Functional studies of key psoriasis-associated genes based on scRNA-seq

Cells were stratified into high- and low-expression groups for each key gene based on median expression (after excluding zero values). Differential expression analysis between these groups was conducted using the standard Seurat workflow, with t-distributed stochastic neighbor embedding (t-SNE) for visualization, applying thresholds of logfc.threshold = 0.3 and min.pct = 0.2. Genes with Benjamini-Hochberg adjusted *P* < 0.05 were subjected to KEGG and GO enrichment analysis; significantly enriched pathways (adjusted *P <* 0.05) were retained. The gene sets corresponding to these significant pathways were extracted, and pathway activity was scored using the AUCell algorithm (v1.20.0). The resulting AUCell activity matrix was analyzed with the limma package to identify pathways with significantly different activities between high- and low-expression groups (adjusted *P <* 0.05). Finally, the differentially activated pathways identified for each of the four key genes were intersected to reveal the convergent pathway alterations driven by their dysregulation.

### Molecular docking

Using the Comparative Toxicogenomics Database (CTD), we screened small-molecule compounds that may downregulate key psoriasis-associated driver genes. The protein products of these key genes were retrieved from the UniProt database. Batch molecular docking was then performed with AutoDock to evaluate the binding between the small-molecule compounds and their target proteins, where lower docking binding energy indicates stronger binding affinity. Finally, the interaction sites between the compounds and proteins were visualized with the open-source version of PyMOL.

### Statistical analysis

All statistical analyses were performed in R. Comparisons among three or more groups were conducted using analysis of variance (ANOVA), while comparisons between two groups were performed with Student*'*s t-test. Correlations were assessed using Pearson correlation coefficients. In the figures, ns denotes not significant; *P < 0.05, **P < 0.01, ***P < 0.001, and ****P < 0.0001. For high-throughput comparisons, multiple testing correction was applied: the Bonferroni correction was used for differential expression analysis, and the Benjamini-Hochberg procedure was used for pathway enrichment, with adjusted *P* < 0.05 considered significant.

## Results

### Single-cell mapping of patients with psoriasis

**Fig 1:**
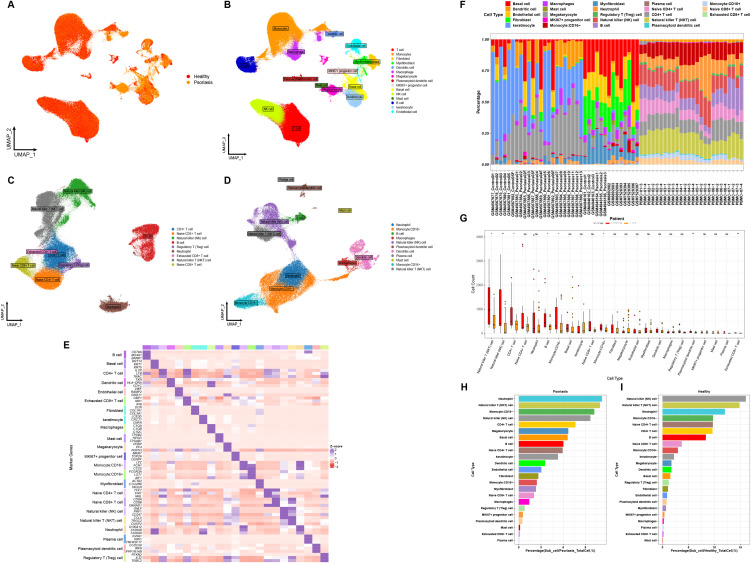
Parsing psoriasis single-cell transcriptional profiles. (A) UMAP projection of healthy and psoriatic samples. (B) First-level UMAP projection and cell type annotation. (C)UMAP projection and annotation of lymphoid lineage immune cells. (D) UMAP projection and annotation of myeloid lineage immune cells. (E) Heatmap showing marker gene expression used for cell type annotation. (F) Proportion of cell types across patients.(G) Differences in cell-type composition between healthy and psoriatic samples. (H, I) Ranking of cell-type abundance in healthy (H) and psoriatic (I) samples.

After data integration and batch-effect correction, we obtained single-cell transcriptomic profiles from 63 samples (psoriatic and healthy), comprising 277,664 cells and 28,189 genes (Fig 1A). Fifteen cell populations were annotated using a semi-supervised approach: T cells, monocytes, fibroblasts, myofibroblasts, dendritic cells, macrophages, megakaryocytes, plasmacytoid dendritic cells, MKI67 + progenitor cells, basal cells, NK cells, mast cells, B cells, keratinocytes, and endothelial cells (Fig 1B).

To gain higher-resolution cellular information, we independently resolved the lymphoid and myeloid immune compartments. The lymphoid lineage was subdivided into nine cell types: CD4 + T cells, naïve CD4 + T cells, NK cells, B cells, regulatory T cells, neutrophils, exhausted CD8 + T cells, NKT cells, and naïve CD8 + T cells (Fig 1C). The myeloid lineage was subdivided into 11 cell types: neutrophils, CD16- monocytes, B cells, macrophages, NK cells, plasmacytoid dendritic cells, dendritic cells, plasma cells, mast cells, CD16 + monocytes, and NKT cells (Fig 1D).

Notably, during sub-clustering we observed a few cross-lineage cell populations: cells annotated as neutrophils appeared transiently in the lymphoid sub-analysis, while cells annotated as B cells, NK cells, and NKT cells were detected in the myeloid sub-analysis. Given that our integrated single-cell matrix was derived from multiple datasets, it is plausible that, despite batch correction, subtle non-biological variation may cause some cells to be transiently redistributed into adjacent clusters during high-resolution subgrouping. Importantly, after completing all sub-analyses, these subgroups were merged and re-integrated to yield a final set of 23 consensus cell types (Fig 1E). This consolidation step resolves the transient cross-lineage ambiguity, as the definitive annotation confirms the identity of all cells based on established marker genes (Fig 1E), ensuring the biological rigor of our final cell-type assignments.

We next compared cell-type abundances across the 63 samples (Fig 1F). A pronounced difference was observed between tissue and peripheral blood samples: tissue samples were dominated by cells of epithelial origin, whereas peripheral blood samples consisted predominantly of immune cells. Comparison between psoriasis and healthy samples (Fig 1G) revealed that megakaryocytes and dendritic cells were significantly more abundant in psoriasis samples, while NK cells and naïve CD8 + T cells were significantly more abundant in healthy samples. The proportions of each cell type in psoriasis and healthy samples are shown in Fig 1H and Fig 1I, respectively; neutrophils exhibited high abundance in both groups. In summary, we integrated multiple single-cell cohorts, resolved single-cell transcriptional profiles in psoriasis, and characterized cell-type composition differences between psoriatic and healthy samples.

### Identification of highly correlated cell types and gene signatures in psoriasis by deconvolution

**Fig 2:**
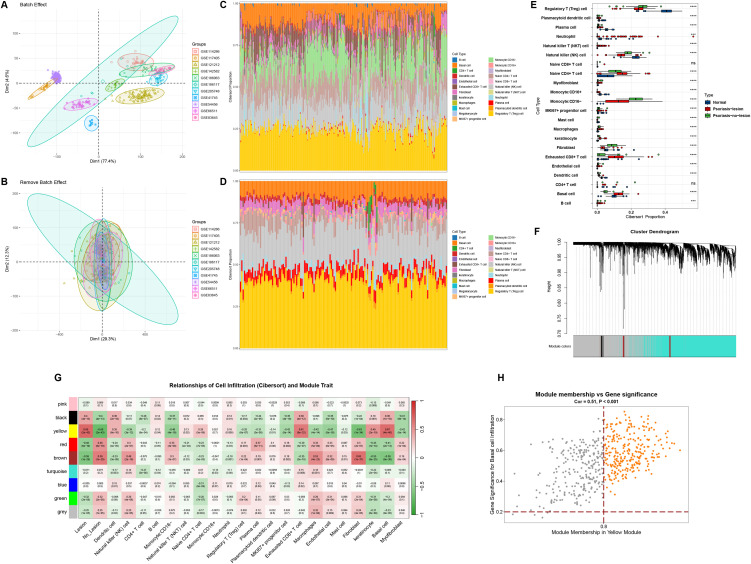
Bulk RNA-seq analysis identifies psoriasis-associated features. Integration of multiple datasets revealed a batch effect (A), which was eliminated after batch correction (B). Cell-type composition was estimated by deconvolution of the healthy cohort (C) and psoriasis cohort (D) in bulk RNA-seq using average cellular expression profiles derived from healthy and psoriatic scRNA-seq datasets.(E) Differences in cell abundance between groups were assessed by ANOVA. ns, not significant; *P < 0.05; **P < 0.01; ***P < 0.001; ****P < 0.0001. (F) WGCNA clustered genes into distinct modules. (G) Pearson correlation analysis of gene modules with psoriatic lesions, non-lesional skin, and cell abundance. (H) Identification of hub genes in the yellow module using thresholds of module membership (MM) > 0.8 and gene significance (GS) > 0.2.

We collected 11 bulk RNA-seq datasets from GEO (Fig 2A), comprising 462 psoriasis-related samples: 231 lesional, 76 non-lesional, and 155 healthy controls. After integration and batch correction, the corrected expression profiles showed successful removal of batch effects across datasets (Fig 2B), and all subsequent analyses were based on these corrected profiles. Using the average expression of the top 50 differentially expressed genes for each cell type from the scRNA-seq data (separately for healthy and psoriasis conditions), we performed CIBERSORT deconvolution to estimate cell-type proportions in the bulk RNA-seq healthy (Fig 2C) and psoriasis (Fig 2D) samples. We observed that exhausted CD8 + T cells and CD16- monocytes were more abundant in psoriasis samples than in healthy samples. This finding was supported by intergroup comparisons (Fig 2E): exhausted CD8 + T cells were significantly higher in lesional psoriasis samples than in the other two groups, while CD16- monocytes were more abundant in non-lesional samples. In addition, basal cells and naïve CD4 + T cells were significantly more abundant in the lesional group than in the other two groups.

To further identify cell types and gene signatures highly correlated with psoriasis, we applied WGCNA to the lesional and non-lesional cohorts (Fig 2F), which partitioned genes into nine color-coded co-expression modules. Correlation analysis between gene modules and cell-type abundances (Fig 2G) revealed that the yellow module was strongly correlated with the psoriasis lesion trait (correlation coefficient = 0.69). Among all cell types, basal cells showed the highest correlation with the yellow module (correlation coefficient = 0.67), suggesting a strong link between basal cells and psoriasis pathogenesis. Hub genes within the yellow module were then identified using thresholds of |module membership (MM)| > 0.8 and |gene significance (GS)| > 0.2, yielding 271 genes highly correlated with both psoriasis lesions and basal cells (Fig 2H).

### Machine learning identifies four key driver genes associated with psoriasis

**Fig 3:**
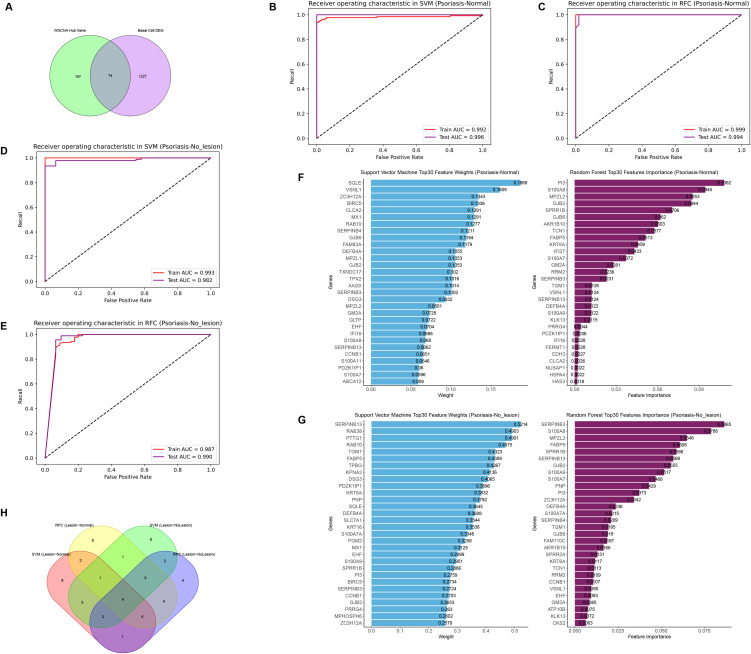
Machine learning-based identification of psoriasis-associated gene signatures. (A) Venn diagram showing the overlap between WGCNA-derived hub genes and differentially expressed genes in psoriatic basal cells. (B, C) ROC curves of the SVM model (B) and RFC model (C) for distinguishing psoriasis from normal samples.(D, E) ROC curves of the SVM model (D) and RFC model (E) for distinguishing psoriatic lesional from non-lesional samples. (F) Top 30 most heavily weighted gene features in the SVM model and top 30 most important gene features in the RFC model for the psoriasis versus normal cohort. (G) Top 30 most heavily weighted gene features in the SVM model and top 30 most important gene features in the RFC model for the lesional versus non-lesional cohort. (H) Intersection of the top 30 features from the four machine learning models.

To refine the relationship between the hub gene set and basal cells, we intersected the basal cell differential genes with the 271 hub genes, obtaining 74 overlapping candidates (Fig 3A). These 74 gene features served as input for SVM and RFC classifiers, using two classification tasks: psoriasis lesion versus non-lesion, and psoriasis lesion versus healthy control.

In the psoriasis versus healthy cohort, the SVM classifier achieved training and validation AUCs of 0.992 and 0.996 (Fig 3B), while the RFC achieved 0.999 and 0.994 (Fig 3C), respectively, demonstrating that both models accurately distinguished psoriasis from normal samples. For the lesional versus non-lesional cohort, the SVM training and validation AUCs were 0.993 and 0.982 (Fig 3D), and the RFC achieved 0.987 and 0.990 (Fig 3E). Although performance on this task was slightly lower, the models still reliably discriminated lesional from non-lesional samples. Overall, the selected gene features exhibited strong discriminatory power across both cohorts, as reflected by the high AUC values

We extracted the top 30 most important features from each of the four models, feature weights for SVM and importance scores for RFC. Fig 3F displays the top 30 features for both classifiers in the psoriasis versus normal cohort, and Fig 3G shows the corresponding rankings for the lesional versus non-lesional cohort. Intersecting these top 30 features from all four models yielded four consensus key genes (Table 1): *DEFB4A*, *GJB2*, *SERPINB3,* and *SERPINB13*. Notably, these genes exhibited consistently high importance scores across all models, underscoring their robustness as candidates.

**Table 1:** The model weights for the four genes were in both cohorts.

Gene	SVM (cohorts1*)	SVM (cohorts2*)	RFC (cohorts1*)	RFC (cohorts2*)
*DEFB4A*	0.1055	0.3608	0.0122	0.0236
*GJB2*	0.1053	0.0844	0.2653	0.5555
*SERPINB3*	0.1002	0.0231	0.2724	0.0865
*SERPINB13*	0.0662	0.0124	0.5214	0.0569

*: cohorts1 was psoriasis and normal cohorts, and cohorts2 was psoriasis focal vs. non-focal cohort.

*DEFB4A* encodes an antimicrobial peptide primarily involved in immune defense and the regulation of inflammatory responses. *GJB2* encodes connexin 26 (Cx26), a gap junction protein that forms intercellular channels, playing a vital role in cell-cell communication and the exchange of small molecules. SERPINB3 and *SERPINB13* belong to the serpin family of serine protease inhibitors. Both encode structurally similar protease inhibitors that function by binding to the active site of target proteases and forming irreversible enzyme-inhibitor complexes.

### Validation of key psoriasis-associated genes

**Fig 4:**
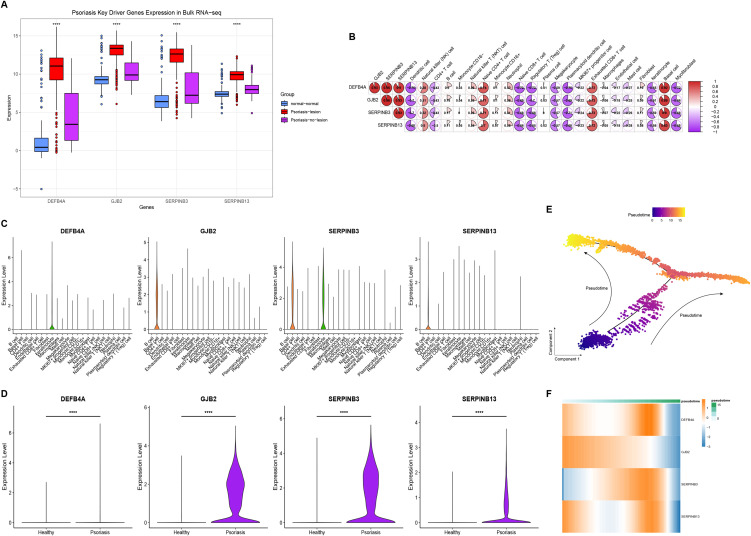
Expression analysis of four key psoriasis driver genes in bulk RNA-seq and scRNA-seq datasets. (A) mRNA expression of the four key driver genes in psoriatic lesional skin, psoriatic non-lesional skin, and normal skin, analyzed by ANOVA. (B) Pearson correlation between the four key driver genes and CIBERSORT-estimated cell fractions. (C) Expression patterns of the four key driver genes across all cell types in the scRNA-seq cohort. (D) Differential expression of the four key driver genes between psoriatic and healthy samples in the scRNA-seq cohort, assessed by t-test. (E) Pseudotime analysis based on basal cells from psoriatic samples to construct a differentiation trajectory. (F) Expression changes of the four key driver genes across different stages of basal cell differentiation. ns, not significant; *P < 0.05; **P < 0.01; ***P < 0.001; ****P < 0.0001.

To confirm the relevance of the four key driver genes to psoriasis, we examined their mRNA expression in bulk RNA-seq datasets. As shown in Fig 4A, all four genes were markedly upregulated in lesional samples, and expression in non-lesional samples was also significantly elevated relative to normal controls. Correlation analysis revealed that *DEFB4A*, *GJB2*, *SERPINB3*, and *SERPINB13* were strongly correlated with basal cells, with correlation coefficients of 0.92, 0.89, 0.90, and 0.83, respectively (Fig 4B). These results indicate that the four genes are highly correlated not only with psoriasis lesions but also with basal cells, largely fulfilling the screening objectives.

We further assessed their expression across all cells in the scRNA-seq cohort (Fig 4C). All four key genes were highly upregulated in basal cells, and *DEFB4A* and *SERPINB3* were additionally upregulated in keratinocytes. This suggests that these genes play critical regulatory roles in cells of epithelial origin. Notably, the four key genes were significantly upregulated in psoriasis, consistent with the bulk RNA-seq findings, further supporting their strong association with the disease.

We next constructed a pseudotime trajectory of basal cell differentiation (Fig 4E) to examine the expression dynamics of the four key genes along this axis. As shown in Fig 4F, all four genes were downregulated at the terminal differentiation stage and were primarily expressed during the early-to-middle and late phases. *DEFB4A* and *SERPINB13* exhibited high expression in both early and late differentiation stages, with reduced expression during an intermediate stable phase, indicating a dynamic expression profile. *GJB2* was predominantly expressed at the pre-differentiation stage, whereas SERPINB3 was predominantly expressed at the post-differentiation stage. These patterns suggest potential associations with distinct differentiation states; however, further functional studies are required to establish their specific roles.

### Pathway Alterations Associated with Key Psoriasis Gene Dysregulation

**Fig 5:**
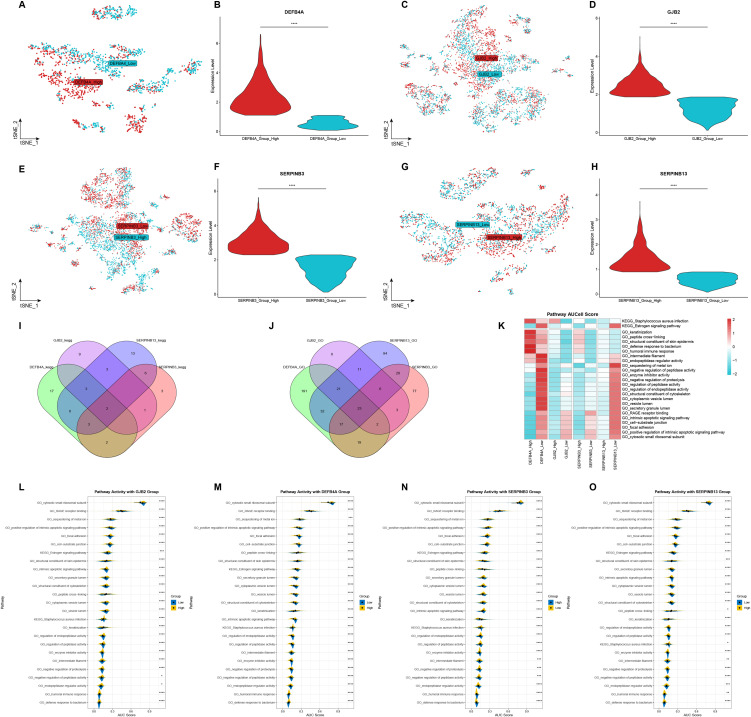
Pathway activity analysis following dysregulation of four key psoriasis driver genes. After stratifying cells into high- and low-expression groups based on the median expression of DEFB4A (A), *GJB2* (B), *SERPINB3* (C), and SERPINB13 (D) in the scRNA-seq cohort, t-SNE visualization was performed for each group.Differential expression between the high- and low-expression groups was further analyzed for *DEFB4A* (E), *GJB2* (F), *SERPINB3* (G), and *SERPINB13* (H). Intersecting *KEGG* (I) and GO (J) pathways with significantly altered activity were identified across the four genes.(K) Heatmap showing the mean AUCell activity of intersecting pathways after stratification by the four genes. (L-O) Comparison of intersecting pathway activity between high- and low-expression groups for *GJB2* (L), *DEFB4A* (M), *SERPINB3* (N), and *SERPINB13* (O). ns, not significant; *P < 0.05; **P < 0.01; ***P < 0.001; ****P < 0.0001.

To investigate how dysregulation of the four key psoriasis genes influences pathway activity in lesional skin and to facilitate the proposal of targeted therapeutic strategies, we conducted a detailed pathway analysis in the scRNA-seq cohort. Cells were stratified into high- and low-expression groups for each gene; t-SNE visualization revealed that, although cells from the two groups partially overlapped, their global transcriptional patterns remained largely distinct (Fig 5A, C, E, G). The key genes were significantly upregulated in their respective high-expression groups (Fig 5B, D, F, H), confirming the presence of meaningful intergroup differences appropriate for downstream analyses.

For each gene, differentially expressed genes were identified between the high- and low-expression groups and subjected to pathway enrichment. Pathway activities were scored and compared to identify those with significantly altered activity. To capture pathways coregulated by all four genes, we intersected the sets of significantly altered pathways across the four comparisons. This yielded two shared KEGG pathways, estrogen signaling pathway and Staphylococcus aureus infection, and 23 shared GO terms, including defense response to bacterium and humoral immune response (Fig 5I, J). Estrogen signaling pathway activity was consistently downregulated in the high-expression groups across all four genes (Fig 5K), indicating that upregulation of these key genes is associated with suppressed estrogen signaling. Because estrogen signaling influences cell and tissue development, growth, differentiation, and function, its suppression may impact processes such as keratinization and cytoskeleton organization; indeed, pathways related to cell differentiation were also significantly altered (Fig 5L-O).

Although the upstream triggers of the four genes*’* upregulation remain unclear, S. aureus infection and humoral immune response pathways were significantly upregulated in the high-expression groups. Notably, elevated *DEFB4A* and *GJB2* expression was associated with markedly increased S. aureus infection pathway activity (Fig 5K). Meanwhile, defense response to bacterium and humoral immune response were significantly upregulated in the *DEFB4A*-high group, consistent with the function of *DEFB4A* as an antimicrobial peptide that defends against bacterial challenge and contributes to host homeostasis. Based on these correlative observations, we propose a hypothesis: upon bacterial challenge (e.g., S. aureus), *DEFB4A* may be overexpressed as a protective antimicrobial response; this upregulation is associated with enhanced humoral immune activity and concomitant suppression of estrogen signaling, which could in turn alter epidermal keratinization and contribute to the formation of psoriatic lesions. Importantly, this is a preliminary, hypothesis-generating model derived from cross-sectional transcriptomic data; causal relationships and the underlying mechanisms require direct experimental validation.

### Preliminary screening of potential binding compounds for key psoriasis genes

Although several treatment modalities for psoriasis are available, including topical therapy and phototherapy, the disease*'*s complex pathogenesis and recurrent flare-ups underscore the urgent need for additional therapeutic options. We previously identified four key psoriasis-associated genes that were significantly upregulated in lesional skin and highly correlated with basal cells. As an initial computational exploration, we combined the Comparative Toxicogenomics Database (CTD) with AutoDock molecular docking to screen for small-molecule compounds that may bind to the encoded proteins.

**Fig 6:**
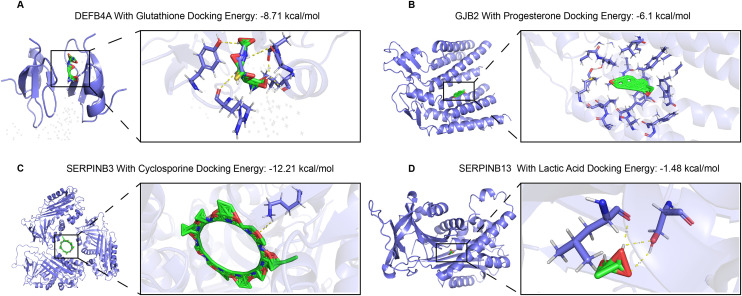
Molecular docking of four psoriasis key driver genes with candidate drugs. (A) Docking of *DEFB4A* with glutathione. (B) Docking of *GJB2* with progesterone. (C) Docking of *SERPINB3* with cyclosporine. (D) Docking of *SERPINB13* with lactic acid.

The docking results indicated that *DEFB4A* and glutathione exhibited a favorable binding energy of −8.71 kcal/mol (Fig 6A). Glutathione, a tripeptide with antioxidant, detoxifying, and immunomodulatory properties, has not yet demonstrated established clinical efficacy in psoriasis. *GJB2* showed a moderate binding energy of −6.1 kcal/mol with progesterone. Progesterone, a steroid hormone essential for endocrine regulation, requires further experimental validation to establish its direct relevance to *GJB2* in psoriasis. Notably, SERPINB3 displayed a very strong binding energy with cyclosporine (−12.21 kcal/mol). Cyclosporine, an immunosuppressant, is an approved psoriasis treatment; this docking result may offer additional structural insight into its interaction with SERPINB3 but does not represent a novel therapeutic strategy per se. For *SERPINB13*, lactic acid yielded a marginal binding energy of −1.48 kcal/mol, a value generally considered too weak for reliable binding; its candidacy should therefore be viewed with caution. Lactic acid, an intermediate in glucose metabolism, is used in some cosmetic and skincare products, but its ability to target *SERPINB13* at pharmacologically relevant concentrations remains speculative.

In summary, this computational screening identified several compounds with varying predicted binding affinities for the four gene products. However, these are preliminary in silico findings and do not constitute validated therapeutic strategies. Further in vitro and in vivo studies are required to evaluate actual binding, functional modulation, and clinical potential.

## Discussion

Psoriasis is a prevalent, non-communicable, and debilitating disorder characterized by chronic pain, disfigurement, and functional impairment, affecting approximately 2–3% of the global population and imposing substantial individual and societal burdens [2–4]. Although no cure exists, identifying key pathogenic genes and developing tailored therapeutic strategies remain urgent priorities. The substantial inter-individual heterogeneity of psoriasis complicates the identification of effective targets and the design of personalized therapies. Single-cell RNA sequencing (scRNA-seq) has emerged as a powerful tool for dissecting this heterogeneity and uncovering distinct cell subpopulations, thereby facilitating the discovery of critical therapeutic targets.

In this study, we integrated single-cell transcriptomic data from 63 psoriatic and healthy samples to construct a comprehensive dataset. Semi-supervised annotation resolved 15 cell taxa into 23 consensus cell populations. Consistent with previous reports, megakaryocytes, dendritic cells, and neutrophils were elevated in psoriasis samples [4,11,12]. Using the average expression of the top 50 differentially expressed genes per cell type, CIBERSORT-based deconvolution was performed to estimate cellular composition. Notably, exhausted CD8 + T cells and naïve CD4 + T cells were more abundant in lesional skin, whereas CD16- monocytes were enriched in non-lesional samples, potentially reflecting immune dysfunction or inflammation subsequent to disease onset [13,14]. These observations align with the established view that psoriasis is driven by T-cell dysregulation [12]. Basal cells also showed greater prevalence in lesional samples. Given the strong association between psoriasis and basal keratinocyte hyperproliferation [4,15,16], this enrichment is consistent with the concept that epidermal hyperplasia and hyperkeratosis, driven by basal keratinocytes and their interplay with T cells and dendritic cells, contribute to disease progression [16].

WGCNA of lesional and non-lesional cohorts identified 271 hub genes significantly associated with both psoriasis lesions and basal cells. Subsequent machine learning classification using SVM and RFC pinpointed four key genes: *DEFB4A* (encoding defensin beta 4A), *GJB2* (encoding connexin 26), *SERPINB3,* and *SERPINB13*. *DEFB4A* encodes an antimicrobial peptide crucial for innate immune defense and inflammatory regulation; it has been implicated in psoriasis and may exert its effects through the IL-17 pathway [17–19]. *GJB2* encodes connexin 26 (Cx26), a gap junction protein that is upregulated in psoriatic plaques and promotes keratinocyte proliferation, and is recognized as a psoriasis susceptibility gene [20–22]. *SERPINB3,* a serine protease inhibitor involved in programmed cell death, serves as a biomarker in autoimmune disorders and cancers and has been confirmed to be upregulated in psoriasis [23–25]. *SERPINB13*, another serpin family member, inhibits cathepsins K and L and has been proposed as a therapeutic target in certain diseases [26,27]. Our findings demonstrate that these four genes are upregulated in psoriatic lesions and share a convergent association with downregulated estrogen signaling pathway activity. Elucidating their functions and coregulated pathways may inform the development of targeted therapeutic strategies.

Computational drug repurposing screening can nominate candidate compounds for further investigation, but the results must be interpreted with caution and require experimental validation. Glutathione displayed a favorable docking pose with DEFB4A. Although glutathione levels are reported to decline in psoriasis [28,29] and N-acetylcysteine (NAC) has been suggested as a precursor [29], the direct therapeutic potential of targeting DEFB4A with glutathione remains unproven. *GJB2* exhibited favorable docking with progesterone, and previous studies indicate that progesterone can downregulate Cx26 mRNA [30,31]. However, whether this interaction is pharmacologically relevant in psoriasis is unknown and warrants dedicated experimental study. Cyclosporine, an established anti-psoriatic drug, showed exceptionally high binding affinity for *SERPINB3* in our docking analysis. This finding does not itself propose a new therapeutic avenue, but rather provides a structural perspective that may complement existing knowledge of cyclosporine*'*s mechanisms. To mitigate its side effects, combination therapies have been explored [32–34], and our data could inform future structure-based optimization. Lactic acid, despite yielding the most favorable docking energy among the screened candidates for *SERPINB13*, exhibited relatively weak binding (−1.48 kcal/mol). Although lactic acid is known for its keratolytic effects [35,36], its potential as a targeted binder of *SERPINB13* is tenuous and would require high-concentration exposure unlikely to be achieved pharmacologically. In summary, our study provides a set of computationally derived candidate compound-target pairs. Any suggestion of a combination regimen targeting these genes is strictly speculative and must be rigorously validated before any clinical consideration.

The novelty of this study lies in the innovative integration of extensive scRNA-seq and bulk RNA-seq datasets, employing advanced algorithms and machine learning techniques to uncover pivotal genes implicated in psoriasis. Extending our investigation to gene function and drug targeting represents another original contribution. Despite these advances, several limitations should be acknowledged. First, although batch correction was applied, residual batch effects may persist. For instance, the cross-lineage cell subsets observed during sub-clustering may reflect residual technical noise and entail annotation uncertainty, even though they were resolved in the final integrated annotations. Future studies should prioritize harmonization of sequencing protocols to ensure data integrity and precision. We also acknowledge that the use of condition-specific CIBERSORT reference matrices may cause estimated cell proportions to partly reflect transcriptional differences rather than pure abundance changes; therefore, cellular abundance comparisons should be interpreted with caution. Additionally, the integrated single-cell cohort combined tissue and peripheral blood samples, which may introduce compositional heterogeneity that influences integration and cell-type comparisons. Furthermore, the 74 input genes for machine learning were pre-selected on the full dataset rather than exclusively on the training set, representing a limitation in maintaining complete training-validation separation during feature selection. Finally, our findings remain in an exploratory and speculative phase, necessitating experimental validation, which constitutes a key focus for future work. Our plans include validating the expression of the identified genes in serum and psoriasis lesion biopsies and investigating their combined therapeutic potential at the cellular and animal levels as a central theme in upcoming studies.

## Conclusions

our analysis identified four key genes, DEFB4A, *GJB2*, *SERPINB3*, and *SERPINB13*, that were significantly upregulated in psoriasis lesions. By examining their roles in regulating pathway activity associated with these lesions, we uncovered potential therapeutic strategies targeting these genes. The screening of small-molecule compounds against these gene products highlights their therapeutic promise in the management of psoriasis.

## Supporting information

S1 Fig Research Route. (PDF)

S1 Table Marker Genes Used for Cell Type Annotation. (XLSX)
