# Supplementary figures and images for: Transcriptomic characterization of key psoriasis-associated genes based on single-cell RNA-seq and machine learning
Source: PLoS One. 2026 Jul 13;21(7):e0352663. doi: 10.1371/journal.pone.0352663 (PMC13362100; doi:10.1371/journal.pone.0352663)

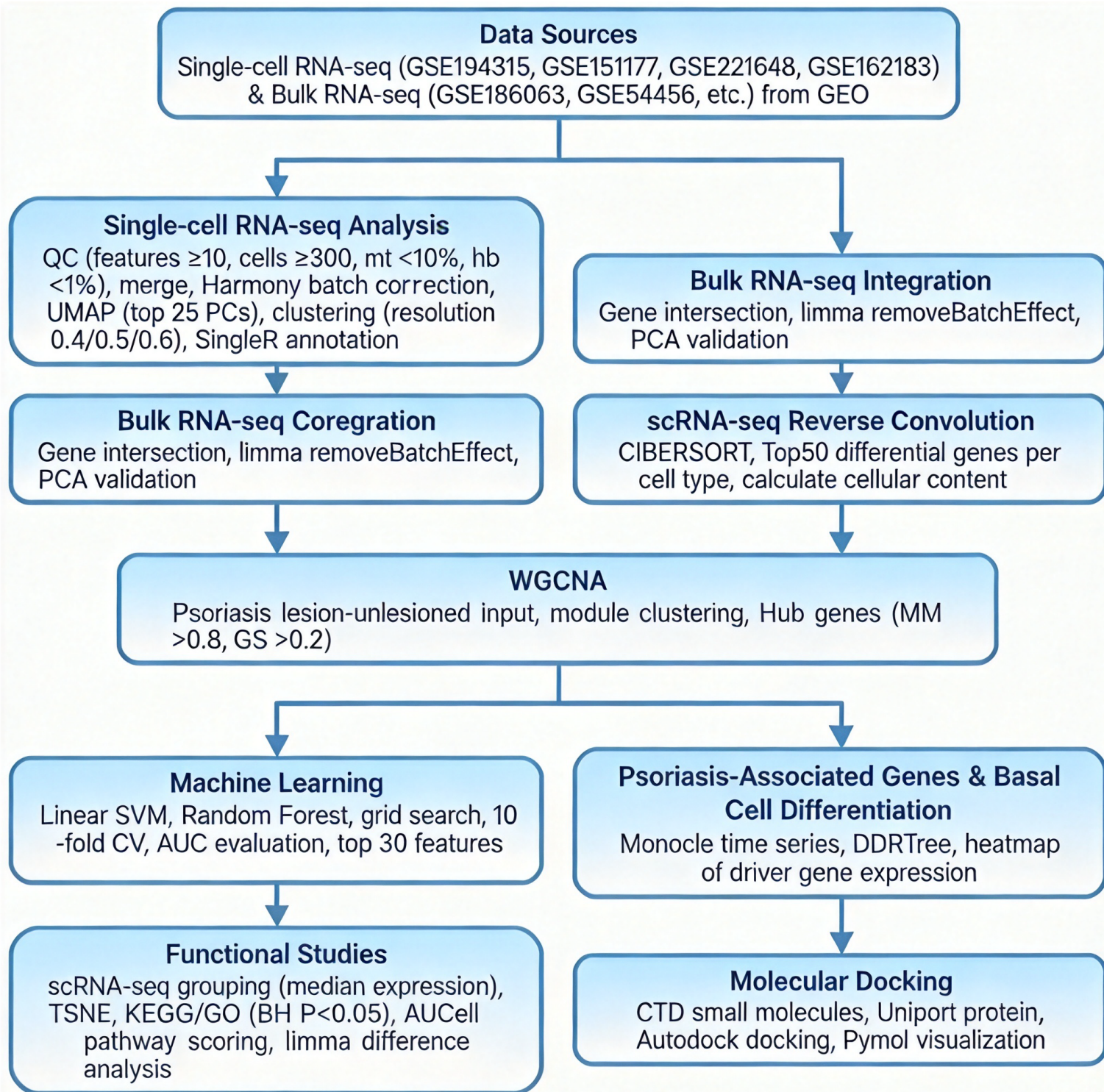

Supplement: S1 Fig — (PDF) [file pone.0352663.s001.pdf]
