# Supplementary material for: Transcriptomic characterization of key psoriasis-associated genes based on single-cell RNA-seq and machine learning
Source: PLoS One. 2026 Jul 13;21(7):e0352663. doi: 10.1371/journal.pone.0352663 (PMC13362100; doi:10.1371/journal.pone.0352663)
Supplement: Attachment — Submitted filename: Response to Reviewers.docx [file pone.0352663.s004.docx]

**Reviewer #1:** This manuscript has several strengths. It addresses a clinically relevant topic by investigating potential driver genes in psoriasis and their possible therapeutic implications. The integration of multiple scRNA-seq and bulk RNA-seq datasets adds value to the study and may improve the robustness of the analysis. The overall workflow is also reasonably comprehensive, incorporating single-cell analysis, deconvolution, WGCNA, machine learning, cross-dataset validation, pathway analysis, and drug-target prediction. In addition, the study provides a relatively detailed view of cellular heterogeneity in psoriasis and links the identified genes to specific cell populations, particularly basal cells. The use of public datasets and the availability of the analysis code further support the transparency and reproducibility of the work.
However, the following three issues should be noted.
**1. The threshold stated in the original text, "each cell needed to contain at least 10 features,"seems somewhat lenient.**

**Response:**

We sincerely thank the reviewer for the careful reading and for pointing out the potential leniency of our QC criteria. Upon re-examining the manuscript and the actual code, we realized that we had inadvertently swapped the descriptions of two key parameters in the earlier version of the Methods section. The actual QC settings we used are as follows:

- min.cells = 10 (genes detected in fewer than 10 cells were removed)
- min.features = 300 (cells with fewer than 300 detected genes were removed)
- percent. mt < 10%
- percent. hb < 1%

These thresholds are in fact comparable to, or more stringent than, the widely used defaults in the scRNA-seq field (e.g., min.cells = 3, min.features = 200, percent. mt < 10-20%). Specifically, our min.features = 300 filter is stricter than the commonly recommended lower bound of 200, and the percent. hb < 1% is a deliberately conservative cutoff adopted to minimize erythrocyte contamination after inspecting the distribution of hemoglobin transcripts in our dataset.

We have now corrected the description in the Methods section of the revised manuscript. We hope this clarification addresses the reviewer’s concern.

**2. The criterion "each feature needed to be expressed in at least 300 cells" seems rather stringent for retaining low-abundance yet biologically meaningful genes.**

**Response:**

We sincerely thank the reviewer for this remark, which further prompted us to re-examine our QC description. We realize that this concern actually originates from the same misstatement described in our response to the previous comment (Comment 1). As clarified above, the actual gene-level filtering threshold we applied was **min.cells = 10,** not 300. The originally worded criterion “expressed in at least 300 cells” was a typographical error that inadvertently swapped the cell-filtering and gene-filtering parameters.

Under the genuine filtering scheme (min. cells = 10), only genes detected in fewer than 10 cells were removed. This is a rather relaxed, widely-adopted default (the Seurat tutorial frequently uses min.cells = 3; a threshold of 5-10 is common for pilot filtering) that is intentionally designed to preserve low-abundance yet biologically meaningful genes. Therefore, we believe our actual parameter does not impose excessive stringency on gene detection.

The corrected description in the Methods section now reads: “Genes detected in fewer than 10 cells were removed.” We hope this clarification resolves the reviewer’s concern.

**3.The authors classified neutrophils under "lymphoid lineage," while B cells, NK cells, and NKT cells appeared under “myeloid lineage.”They explained this using “The explanation that these populations were ‘sandwiched between’ clusters and that this was hypothesized to result from cell-cell interactions,” but this interpretation seems somewhat subjective.**

**Response:**

We sincerely thank the reviewer for highlighting this critical point. Upon reflection, we fully agree with the reviewer that attributing this cross-lineage contamination to cell-cell interactions was an over-interpretation and is, as pointed out, unduly subjective.

As the reviewer suggested, we have now reconsidered the origin of these minor heterogeneous clusters. We believe a more probable and conservative explanation stems from our analytical pipeline. In our study, the total single-cell matrix was merged from multiple samples (data sets). Despite batch-effect correction, residual technical noise can persist. In these fine-grained sub-clustering analyses, such noise can occasionally manifest as a small number of one lineage’s cells being pulled into the sub-clustering space of another lineage due to variable capture efficiency or transcriptomic similarity at low-dimensional projections. These are transient analytical artifacts.

Crucially, as described in our workflow, the final step after all sub-clustering was to re-merge and integrate all populations to obtain a unified, definitive cell atlas (total 23 cell types, Fig.1E). This final merging step effectively corrects these artifacts, as cells are re-annotated and collapsed into their true major lineage groups, ensuring that no neutrophils remain mislabeled as lymphoid, nor lymphoid cells as myeloid, in the final results presented to the reader. Our original description failed to adequately communicate this and instead offered an unsupported biological speculation. We have, therefore, comprehensively revised the relevant section in the manuscript to articulate this more cautious, data-processing-centric rationale. We hope the reviewer finds this revised interpretation more appropriate.

**Reviewer #2:** This study integrates multiple public single-cell RNA-seq and bulk RNA-seq datasets to identify key driver genes in psoriasis. Using WGCNA and machine learning (SVM and RFC), the authors identified four genes—DEFB4A, GJB2, SERPINB3, and SERPINB13—as potential therapeutic targets, followed by molecular docking to screen for small molecule compounds. The topic has translational potential and the four identified genes have prior literature support. However, the manuscript suffers from logical gaps, insufficient methodological transparency, overinterpretation of preliminary findings, and language issues that compromise reproducibility and clarity.

Recommendation: Major Revision

Major Issues
**1. The role and identification of “basal cells” are not adequately established**
The abstract and results emphasize a correlation between hub genes and basal cells, yet basal cells are not introduced in the Background, nor is their functional relevance to psoriasis explained. The Methods do not specify how basal cells were defined or isolated from the scRNA-seq data (e.g., which marker genes were used). Without this foundation, the reader cannot assess why basal cells are central to the analysis or whether the identification was appropriate.

What needs to be addressed: The authors must clarify the biological rationale for focusing on basal cells in the Background, and provide a clear, reproducible definition of how basal cells were identified in the scRNA-seq dataset.

**Response:**

We sincerely thank the reviewer for this constructive suggestion. We fully agree that the original manuscript lacked proper contextualization and a reproducible definition of basal cells, which may have obscured the rationale of our study.

- Background revision:

As advised, we have now added a brief statement in the Introduction to establish the biological relevance of basal cells to psoriasis. Specifically, we note that basal cells, as the proliferative keratinocyte population residing in the basal layer of the epidermis, are widely recognized to play a pivotal role in the hyperproliferation and aberrant differentiation characteristic of psoriatic epidermis. This provides the necessary foundation for why this cell population became the focus of our downstream investigation.

- Identification of basal cells in the scRNA-seq data:

We would like to clarify that basal cells were not pre-selected based on a targeted isolation strategy. Instead, they were identified a posteriori from the full, unbiased single-cell transcriptomic landscape. After standard clustering analysis, differentially expressed genes were calculated for each cluster, and the cluster corresponding to basal cells was annotated using a panel of established keratinocyte lineage markers. To ensure full transparency and reproducibility, we have now added a supplementary table (Supplementary Table 1) listing the key markers used to define basal cells. Additionally, the Methods section now explicitly states this annotation pipeline.

We believe these additions adequately address the reviewer’s concerns by providing both the biological context and a clear, reproducible definition of the basal cells that underpin the study’s conclusions.

**2. The filtering process from WGCNA hub genes to machine learning input is not transparent**
The Methods state that 271 hub genes were identified by WGCNA (MM > 0.8, GS > 0.2). The Results then mention that these were intersected with “basal cell differential genes” to obtain 74 genes used for machine learning. This critical intermediate step is missing from the Methods section, making the analysis workflow incomplete and non-reproducible.

What needs to be addressed: The authors must describe in the Methods how the 74-gene set was derived, including the criteria used to define “basal cell differential genes” (e.g., fold-change and significance thresholds). The complete analytical pipeline should be transparent.

**Response:**

We thank the reviewer for this valuable suggestion. The omission of the criteria used to define “basal cell differential genes” and the description of how hub genes were intersected with them was indeed a significant oversight in our earlier manuscript. We have now added a complete description of this process in the Methods section. Specifically:

Basal cell differential gene definition: We identified genes differentially expressed between basal cells and all other cell types using the FindMarkers function in Seurat. The criteria were: only positive markers (only.pos = TRUE), expressed in at least 10% of basal cells (min.pct = 0.1), average log2 fold-change > 0.25 (logfc.threshold = 0.25), and adjusted P-value < 0.05.

Intersection: The 271 hub genes were intersected with the basal cell differential gene set, resulting in 74 overlapping genes (Fig. 3A), which were then advanced to the machine learning analysis.

The Methods section has been revised accordingly to include this detailed pipeline, ensuring full transparency and reproducibility.

**3. The selection of the four key driver genes lacks justification**
The manuscript states that the top 30 features from each of the four machine learning models were intersected to yield four genes. However, no rationale is provided for choosing “top 30” as the cutoff. Moreover, the importance or weight values for the four genes are not reported, leaving the reader unable to evaluate whether these genes were consistently top-ranked across models or whether the cutoff was arbitrary.

What needs to be addressed: The authors should justify the feature selection threshold (e.g., by showing cumulative importance or stability across cross-validation folds) and provide the specific importance/weight values for the four genes across all models. This will allow assessment of the robustness of the selection.

**Response:**

We sincerely thank the reviewer for this important comment. We fully agree that the choice of “top 30” needs a clear rationale, and that the feature importance values should be transparently reported.

- Justification of the “top 30” feature cutoff

The threshold of top 30 features was selected to strike a balance between capturing the most informative features and reducing noise from low-importance variables. In high-dimensional biological datasets, importance rankings often plateau after the first few dozen features, and retaining a moderate number of features is a pragmatic strategy commonly adopted in similar multi-model feature selection pipelines (e.g., selecting top 10–50 features per model). For our analysis, we tested the stability of the final gene set by varying the cutoff from 20 to 50; the four selected genes (DEFB4A, GJB2, SERPINB3 and SERPINB13) remained stably in the intersection across a wide range, indicating that the selection is not sensitive to the exact cutoff. We have now added this justification to the Methods section.

- Feature importance values of the four key genes

As suggested, we have now provided the specific importance scores (weights) for the four genes across all four machine learning models in **Table 1**. This pattern confirms that the four genes were not marginal selections driven by an arbitrary cutoff, but rather consistently dominant across models. We believe this transparency fully addresses the reviewer’s concern regarding the robustness of our selection.

**4. The molecular docking findings are overinterpreted as validated therapeutic strategies**
The docking results are presented as if they constitute validated therapeutic strategies, but several compounds have weak clinical evidence (e.g., glutathione, lactic acid) or marginal binding energy (e.g., -1.48 kcal/mol for lactic acid). While cyclosporine is a known psoriasis treatment, its docking with SERPINB3 does not in itself establish a new therapeutic approach. Presenting these preliminary in silico findings as “targeted therapeutic strategies” overstates the conclusions.

What needs to be addressed: The conclusions must be revised to reflect the preliminary nature of the docking results. The authors should clearly differentiate between well-established drugs (e.g., cyclosporine) and speculative candidates, and avoid claiming therapeutic strategies without experimental validation.

**Response:**

We sincerely thank the reviewer for this crucial remark. We fully agree that our original presentation overinterpreted the computational docking results by framing them as validated therapeutic strategies. This was an inappropriate conflation of in silico screening with clinical applicability, and we apologize for the overstatement.

In response, we have comprehensively revised the Results and Discussion sections to reflect the strictly preliminary nature of these findings. Specifically:

- We have replaced the phrase “targeted therapeutic strategies” throughout with terms such as “potential candidate compounds” or “preliminary screening of binding compounds,” and made clear that these represent the first step of a discovery pipeline, not clinically actionable strategies.
- We now clearly distinguish between drugs with established clinical evidence in psoriasis (e.g., cyclosporine) and compounds that currently lack such evidence (e.g., glutathione, lactic acid, progesterone). For cyclosporine, we note that its docking with SERPINB3 provides supplementary structural insight but does not itself define a new therapeutic direction; for the other compounds, we explicitly state that their clinical relevance to psoriasis is speculative and requires validation.
- We have added qualifying language in the Discussion, emphasizing that all in silico results require experimental confirmation and that binding energy alone, particularly marginal values such as the -1.48 kcal/mol for lactic acid, is insufficient to propose clinical utility.
- The revised sections now consistently use cautious language (e.g., “screened compounds,” “potential binders,” “warrants further investigation”) and avoid implying that the reported docking data constitute a ready-to-use therapeutic regimen.

We believe these revisions address the reviewer’s concern by accurately calibrating the strength of our claims to the in silico nature of the evidence. The full modified text is provided in the revised manuscript with all changes highlighted.

**5. Multiple testing correction is not addressed**
Multiple comparisons are reported throughout (e.g., differential gene expression, pathway enrichment) using nominal P-values without mention of correction for false discovery rate. This raises concerns about inflated type I error rates.

What needs to be addressed: The authors must specify in the Statistical Analysis section whether and how multiple testing was corrected (e.g., Benjamini-Hochberg). If corrections were applied, adjusted P-values should be reported; if not, the authors should explain why and acknowledge this limitation.

**Response:**

We thank the reviewer for emphasizing this critical statistical point. We agree that failure to mention multiple testing correction could raise concerns about type I error inflation. In our analysis, multiple testing correction was indeed applied for the high-dimensional comparisons where it is essential:

- Differential gene expression analysis: Differentially expressed genes were identified using the FindMarkers function in Seurat, which by default applies the Bonferroni correction. The reported significant genes were filtered using the adjusted P-value (e.g., adjusted P < 0.05). We have now clarified this in the Methods.
- Pathway enrichment analysis: For over-representation analysis (e.g., GO/KEGG enrichment), we applied the Benjamini-Hochberg (BH) procedure to control the false discovery rate. Enriched terms were considered significant at an adjusted P-value (FDR) < 0.05. This has been added to the revised Methods.
- Other comparisons: For standard hypothesis tests (e.g., two-group t-test, ANOVA, Pearson correlation) performed on a limited number of pre-defined comparisons, nominal P-values are reported, which is common practice in the field when the number of tests is small and exploratory.

We have now added a sentence in the Statistical Analysis section explicitly stating that multiple testing corrections were applied.

Minor Issues
**6. Writing quality and formatting consistency**
The manuscript contains non-standard terminology, informal phrasing, and inconsistent formatting (e.g., figure citation styles, italicization of P-values). These issues detract from readability and professionalism.

What needs to be addressed: The authors should engage a native English speaker or professional editing service to improve language clarity and scientific tone. Formatting (figure references, statistical notation) should be standardized throughout.

**Response:**

We sincerely appreciate the reviewer’s constructive comment on the writing quality and formatting. In response, we have thoroughly revised the manuscript to address these issues. The text has been professionally edited by a native English-speaking expert with scientific editing experience to improve clarity, tone, and adherence to standard scientific terminology. Additionally, we have carefully standardized the formatting throughout the manuscript, including figure citation styles, italicization of P-values, and consistency of statistical notation. We believe these revisions have significantly enhanced the readability and professionalism of the manuscript. All changes are highlighted in the revised manuscript for the reviewer’s convenience.

**7. Data availability statement requires revision**
The statement includes “or available from the corresponding author upon request,” which is not consistent with PLOS’s open data policy. All data used are from public GEO datasets.

What needs to be addressed: The data availability statement should be revised to state that all data are publicly accessible, with accession numbers provided, and remove the “upon request” option.

**Response:**

We thank the reviewer for pointing out this inconsistency with PLOS’s open data policy. We have now completely revised the Data Availability Statement. The original phrasing “or available from the corresponding author upon request” has been removed. Furthermore, to ensure full transparency and reproducibility, all intermediate data generated during this study, including processed gene expression matrices, differential gene lists, and machine learning inputs, have been deposited in a public repository and are accessible via https://github.com/Jyyy9689/Psoriasis. These changes bring the statement into full compliance with PLOS’s open data policy and ensure that all data are freely available without restrictions.

**Reviewer #3**: This study brings together multiple cohorts and combines single-cell analysis, deconvolution, WGCNA, machine learning, and functional enrichment to identify psoriasis-associated candidate genes and explore their possible biological relevance. There is clearly a substantial amount of work here, and the overall analytical framework is fairly complete. One of the stronger parts of the paper is the use of WGCNA to connect the cell-related findings with the later gene-prioritization steps, which helps the different parts of the analysis hold together. The study therefore provides some potentially useful information on psoriasis-associated genes. Even so, there are still several concerns about methodological rigor, clarity of presentation, and the extent to which some of the interpretations are supported by the data. For this reason, I think the manuscript would need major revision before further consideration.

**1. The scRNA-seq QC thresholds need to be checked more carefully.** The requirement that each cell contain at least 10 features is far below. It is usually used in single-cell quality control and would not effectively remove low-quality cells. At the same time, requiring each feature to be expressed in at least 300 cells seems unusually stringent and could remove subpopulation-specific genes or markers from rare cell types. These two thresholds are difficult to understand and may reflect either incorrect parameter reporting or insufficient methodological description. A clearer explanation of the actual settings and the rationale for them would be helpful.

**Response:**

We sincerely thank the reviewer for this careful observation. The seemingly contradictory QC thresholds indeed resulted from an inadvertent parameter-swapping error in the original manuscript. Upon re-examination of our analysis code, we confirmed that the actual filtering parameters used were:

- min.features = 300 (cells with fewer than 300 detected genes were removed)
- min.cells = 10 (genes detected in fewer than 10 cells were removed)
- percent.mt < 10%
- percent.hb < 1%

Thus, the cell-level filter (≥300 genes) is in fact stricter than the widely used default of 200, which effectively removes low-quality cells, while the gene-level filter (≥10 cells) is only moderately more conservative than common defaults (e.g., 3 cells) and intentionally preserves rare cell-type markers. This resolves the apparent contradiction: our actual thresholds are not simultaneously too lax and too stringent. We have now thoroughly corrected the description in the Methods section of the revised manuscript. We hope this clarification satisfactorily addresses the reviewer’s concern.

**2. The construction of the CIBERSORT reference matrix is not entirely clear.** The phrase “Top50 differential genes for each cell subpopulation in psoriasis and healthy samples” can be read in more than one way, and it is hard to tell whether these genes are intended to be cell-type-specific signature genes or genes identified by comparing psoriasis and healthy samples within each cell subpopulation. If the reference was indeed based on within-cell-subpopulation psoriasis-versus-healthy differential genes rather than conventional cell-type-specific signatures, this should probably be acknowledged in the limitations, because such a strategy could mix disease-state signals into the deconvolution and make the estimated cell fractions harder to interpret.

**Response:**

We thank the reviewer for this insightful comment. We have now clarified the reference matrix construction process. Specifically, the signature matrices were built separately for healthy and psoriasis samples: for each group, the top 50 cell-type-specific marker genes (up-regulated relative to all other cells within that group) were identified, and their average expression per cell type was used as the reference. This group-specific approach was originally adopted because psoriasis may alter the transcriptional profiles of skin cells, and we aimed to improve deconvolution accuracy by capturing the disease-specific cellular phenotypes. However, as the reviewer correctly notes, this could introduce disease-state signals into the cell proportion estimates, making it difficult to disentangle true cellular abundance changes from transcriptional alterations. We have now explicitly described this strategy in the Methods and have added a careful discussion of this limitation in the revised manuscript (please see the Discussion section ). We believe this addresses the reviewer’s concern.

3. In the further subdivision of lymphoid and myeloid cells, several apparently cross-lineage cell types were reported. The manuscript mainly attributes this to lineage-feature mixing caused by cell-cell interactions, but I am not sure the current evidence is sufficient to support such a large interpretive leap. Annotation accuracy, subclustering purity, and the possibility of doublets seem equally important here. At minimum, this issue should be discussed more cautiously, and some uncertainty in these subtype annotations should be acknowledged.

**Response:**

We thank the reviewer for this important point. We fully agree that attributing cross-lineage cells to cell-cell interactions was an over-interpretation not supported by sufficient evidence. In the revised manuscript, we have replaced this biological speculation with a more conservative, data-processing-centric explanation. Specifically, we now discuss that these transient, minor cross-lineage clusters likely arise from residual technical noise, e.g., imperfect batch correction after merging multiple single-cell datasets, or local transcriptomic similarity at low-dimensional projections. Importantly, as we clarify in the revised text, these cells were not retained as a distinct cross-lineage group in the final analysis; after all sub-analyses, the populations were merged and re-integrated into the final 23 consensus cell types, which resolved any transient annotation ambiguity and ensured that all cells were correctly assigned to their bona fide lineage identities. We have also acknowledged the inherent uncertainty in fine subclustering annotations in the revised Discussion. We hope this more cautious interpretation addresses the reviewer’s concern.

4. The integrated single-cell cohort includes both tissue-derived and peripheral-blood-derived samples. It naturally differ quite substantially in cellular composition. Similar designs do exist in the literature, so this is not inherently unreasonable, but the manuscript does not fully explain whether downstream analyses were stratified by sample source or otherwise controlled for it. Because this kind of heterogeneity could affect single-cell integration, cell-composition comparisons, and later interpretation, it would be better to state this more explicitly, at least in the limitations.

**Response:**

We thank the reviewer for this practical observation. We have added a sentence in the Methods clarifying that, to capture a comprehensive spectrum of cellular features, the integrated dataset was used globally for downstream analyses without stratification by sample source. In addition, we acknowledge in the Discussion that the inherent compositional heterogeneity of mixing tissue and blood samples may influence integration and cell-type comparisons, and should be considered when interpreting the results.

5. There also seems to be an unedited residual sentence in the Methods section on cell annotation, for example “method for cellular annotation of cell clusters.” This should simply be cleaned up.

**Response:**

We thank the reviewer for catching this oversight. This was indeed an unedited residual phrase left over from drafting. We have now removed it and carefully rechecked the entire Methods section to eliminate any similar editing artifacts.

6. The machine-learning workflow would benefit from a more explicit description. The manuscript mentions train/validation split, grid search, and cross-validation, but it is still not clear whether all of these steps were carried out strictly within the training set. It would be useful to state clearly whether the 74 candidate genes were selected only within the training data, whether grid search and 10-fold cross-validation were restricted to the training set, and whether extraction of the top 30 features was fully isolated from the validation set. If not, that should at least be acknowledged as a limitation.

**Response:**

We thank the reviewer for this rigorous methodological comment. We have now revised the machine learning description to clearly specify which steps were performed within the training set and which were not. Specifically:

- The 74 candidate genes were derived from the intersection of WGCNA hub genes and basal cell differential genes using the full dataset, not exclusively from the training set. We acknowledge this as a limitation, as it means that feature pre-selection was not fully isolated from the validation data.
- Grid search for hyperparameter tuning and 10-fold cross-validation were both strictly confined to the training set.
- Feature importance extraction (top 30 features per model) was also performed solely on the training set; the validation set was held out completely until final model evaluation.

We have updated the Methods section with this explicit breakdown and added a sentence in the Discussion acknowledging that the pre-selection of the 74-gene input set on the full dataset represents a limitation with respect to the ideal of complete training–validation isolation.

7. The sentence “extremely strong predictive performance, which is closely related to the importance and weight of the traits” does not read like a standard machine-learning interpretation. The current wording makes the high AUC sound as though it can be directly explained by the “importance and weight of the traits,” which is not very precise. It would read more naturally to say that the selected gene features showed strong discriminatory power.

**Response:**

We agree and have revised the sentence to state that the selected gene features demonstrated strong discriminatory power, making the interpretation more precise and aligned with standard machine-learning terminology.

8. The phrase “gene inlinearity” in the WGCNA section is unusual and likely to confuse readers. Something like “based on gene co-expression patterns” or “based on gene expression correlations” would be much clearer.

**Response:**

We thank the reviewer for pointing this out. We have replaced "gene inlinearity" with "gene co-expression patterns," which accurately reflects the WGCNA methodology and is more accessible to readers.

9. The pseudotime interpretation for basal cells also feels a little too strong in places. What the current results support more directly is that these key genes show dynamic expression across different differentiation stages. That is not quite the same as showing that they “play a regulatory role” or are specifically related to basal-cell development or senescence. Since this is not the central focus of the paper, a more restrained wording would probably make the manuscript feel more rigorous rather than less.

**Response:**

We fully agree and have revised the pseudotime interpretation throughout. We now describe only the observed dynamic expression patterns of the four genes across the inferred differentiation trajectory, removing speculative claims about regulatory roles, development, or senescence. We believe this more restrained wording improves the rigor of the manuscript .

10. The repeated use of “driver genes” throughout the manuscript may also be stronger than the current evidence allows. In many contexts, “driver” implies a more direct causal or functionally driving role, whereas the present study mainly relies on in silico analyses .So more neutral alternatives such as “key genes,” “candidate genes,” or “psoriasis-associated genes” may be more appropriate unless direct functional validation is added.

**Response:**

We fully agree with the reviewer’s concern. We have removed the term “driver genes” throughout the manuscript and replaced it with more neutral alternatives such as “key genes” or “candidate genes,” consistent with the in silico nature of our current evidence.

11. The latter half of the section on altered pathway activity in Results reads much more strongly than the earlier, more results-oriented parts of the paper. The discussion around Staphylococcus aureus infection, DEFB4A, and estrogen signaling moves rather quickly from pathway-level association to a fairly complete upstream-trigger/downstream-pathogenesis narrative. This part would be more convincing if it were framed more explicitly as hypothesis-generating.

**Response:**

We thank the reviewer for this guidance. We have carefully revised the relevant Results section to remove deterministic language and now explicitly frame the pathway-level interpretation as hypothesis-generating. The revised text focuses on the observed associations and clearly states that the proposed upstream-trigger/downstream-pathogenesis narrative is a working hypothesis requiring future experimental validation.

12. The molecular docking analysis is better treated as a supplementary component rather than a strong part of studies of this type. Some of the drug-related interpretations are written too firmly, and the wording in the Discussion and Conclusion around “therapeutic strategies” and “therapeutic promise” would be better toned down.

**Response:**

We fully agree and have addressed this point in our earlier revisions. The molecular docking analysis is now presented as a supplementary computational screening component, and all drug-related interpretations have been substantially toned down. The terms “therapeutic strategies” and “therapeutic promise” have been removed or replaced with cautious language emphasizing the preliminary, hypothesis-generating nature of the findings.

13. Because of so many analytical modules are integrated in this study, a simple workflow schematic would make the paper much easier to follow. This is important for showing how the analysis moves.

**Response:**

We thank the reviewer for this helpful suggestion. We have now added a workflow schematic as Supplementary Fig. 1 to provide a clear overview of the analytical pipeline.

14. The readability of Fig. 1F could be improved. The connection between the bars and the sample labels below is not very clear, which makes the figure a bit hard to follow. It would help to adjust the layout so that each sample label lines up more clearly with its corresponding bar.

**Response:**

We appreciate this suggestion. We have revised Fig. 1F to improve readability. The layout has been adjusted so that each sample label now aligns more clearly with its corresponding bar, making the figure easier to follow.

15. The current Data Availability Statement may still need some strengthening to better align with the PLOS ONE data policy. Providing the GEO accession numbers and the analysis code is helpful, but if the GitHub repository mainly contains code rather than the processed outputs behind the main figures and statistical analyses, it is still unclear whether all data needed to reproduce the study’s findings are publicly available without restriction. For a study of this type, it would be helpful to make the main source data publicly available as well, including at least the cell-annotation results, sample-level cell proportion tables, CIBERSORT outputs and reference-gene lists, WGCNA module and hub-gene tables, the 74 intersecting genes and top feature lists from the machine-learning analysis, differential gene and enrichment results, AUCell-related outputs, pseudotime-related results, and docking summary tables. In addition, “available from the corresponding author upon request” does not seem fully consistent with the PLOS ONE policy unless specific restrictions are clearly stated. It would therefore be helpful to revise the Data Availability Statement and indicate more clearly where these processed datasets can be accessed.

**Response:**

We thank the reviewer for this detailed guidance. In response, we have further strengthened the Data Availability Statement and the public repository content. The GitHub repository (https://github.com/Jyyy9689/Psoriasis) now contains not only the analysis code, but also all processed data files required to reproduce the findings, including: cell annotation results, sample-level cell proportion tables, CIBERSORT outputs and reference gene lists, WGCNA module assignments and hub gene tables, the 74 intersecting genes and top feature lists from machine learning, differential expression and pathway enrichment results, AUCell-related outputs, pseudotime results, and molecular docking summary tables. The “available upon request” option has been completely removed, and the revised Data Availability Statement now specifies that all data are publicly accessible without restriction, in full compliance with the PLOS ONE policy.
