# Supplementary material for: Transcriptomic characterization of key psoriasis-associated genes based on single-cell RNA-seq and machine learning
Source: PLoS One. 2026 Jul 13;21(7):e0352663. doi: 10.1371/journal.pone.0352663 (PMC13362100; doi:10.1371/journal.pone.0352663)
Supplement: Peer review history

# Peer review: Decision Letter 0

**DOI:** 10.1371/journal.pone.0352663.r001

**Authors:** Zhengrui Li

29 Mar 2026

PONE-D-26-09632

Identifying key psoriasis driver genes and constructing targeted therapeutic strategies based on single-cell RNA-seq and machine learning

PLOS One

Dear Dr. Xu,

Thank you for submitting your manuscript to PLOS ONE. After careful consideration, we feel that it has merit but does not fully meet PLOS ONE’s publication criteria as it currently stands. Therefore, we invite you to submit a revised version of the manuscript that addresses the points raised during the review process.

Please submit your revised manuscript by May 13 2026 11:59PM. If you will need more time than this to complete your revisions, please reply to this message or contact the journal office at plosone@plos.org. When you're ready to submit your revision, log on to https://www.editorialmanager.com/pone/ and select the 'Submissions Needing Revision' folder to locate your manuscript file.

Please include the following items when submitting your revised manuscript:

- A letter that responds to each point raised by the academic editor and reviewer(s). You should upload this letter as a separate file labeled 'Response to Reviewers'.
- A marked-up copy of your manuscript that highlights changes made to the original version. You should upload this as a separate file labeled 'Revised Manuscript with Track Changes'.
- An unmarked version of your revised paper without tracked changes. You should upload this as a separate file labeled 'Manuscript'.

If you would like to make changes to your financial disclosure, please include your updated statement in your cover letter. Guidelines for resubmitting your figure files are available below the reviewer comments at the end of this letter.

If applicable, we recommend that you deposit your laboratory protocols in protocols.io to enhance the reproducibility of your results. Protocols.io assigns your protocol its own identifier (DOI) so that it can be cited independently in the future. For instructions see: https://journals.plos.org/plosone/s/submission-guidelines#loc-laboratory-protocols. Additionally, PLOS ONE offers an option for publishing peer-reviewed Lab Protocol articles, which describe protocols hosted on protocols.io. Read more information on sharing protocols at https://plos.org/protocols?utm_medium=editorial-email&utm_source=authorletters&utm_campaign=protocols.

As the corresponding author, your ORCID iD is verified in the submission system and will appear in the published article. PLOS supports the use of ORCID, and we encourage all coauthors to register for an ORCID iD and use it as well. Please encourage your coauthors to verify their ORCID iD within the submission system before final acceptance, as unverified ORCID iDs will not appear in the published article. *Only*  the individual author can complete the verification step; PLOS staff *cannot*  verify ORCID iDs on behalf of authors.

We look forward to receiving your revised manuscript.

Kind regards,

Zhengrui Li

Academic Editor

PLOS One

**Journal Requirements:**

When submitting your revision, we need you to address these additional requirements.

1. Please ensure that your manuscript meets PLOS ONE's style requirements, including those for file naming. The PLOS ONE style templates can be found at

https://journals.plos.org/plosone/s/file?id=wjVg/PLOSOne_formatting_sample_main_body.pdf and

https://journals.plos.org/plosone/s/file?id=ba62/PLOSOne_formatting_sample_title_authors_affiliations.pdf

2. Please note that PLOS One has specific guidelines on code sharing for submissions in which author-generated code underpins the findings in the manuscript. In these cases, all author-generated code must be made available without restrictions upon publication of the work. Please review our guidelines at https://journals.plos.org/plosone/s/materials-and-software-sharing#loc-sharing-code and ensure that your code is shared in a way that follows best practice and facilitates reproducibility and reuse.

3. In the online submission form you indicate that your data is not available for proprietary reasons and have provided a contact point for accessing this data. Please note that your current contact point is a co-author on this manuscript. According to our Data Policy, the contact point must not be an author on the manuscript and must be an institutional contact, ideally not an individual. Please revise your data statement to a non-author institutional point of contact, such as a data access or ethics committee, and send this to us via return email. Please also include contact information for the third party organization, and please include the full citation of where the data can be found.

4. Please note that your Data Availability Statement is currently missing a direct link to access each database. If your manuscript is accepted for publication, you will be asked to provide these details on a very short timeline. We therefore suggest that you provide this information now, though we will not hold up the peer review process if you are unable.

6. Your ethics statement should only appear in the Methods section of your manuscript. If your ethics statement is written in any section besides the Methods, please move it to the Methods section and delete it from any other section. Please ensure that your ethics statement is included in your manuscript, as the ethics statement entered into the online submission form will not be published alongside your manuscript.

7. Please upload a new copy of Figure 5 as the detail is not clear. Please follow the link for more information:  https://journals.plos.org/plosone/s/figures

8. If the reviewer comments include a recommendation to cite specific previously published works, please review and evaluate these publications to determine whether they are relevant and should be cited. There is no requirement to cite these works unless the editor has indicated otherwise.

[Note: HTML markup is below. Please do not edit.]

Reviewers' comments:

Reviewer's Responses to Questions

**Comments to the Author**

1. Is the manuscript technically sound, and do the data support the conclusions?

The manuscript must describe a technically sound piece of scientific research with data that supports the conclusions. Experiments must have been conducted rigorously, with appropriate controls, replication, and sample sizes. The conclusions must be drawn appropriately based on the data presented.

Reviewer #1: Yes

Reviewer #2: Partly

Reviewer #3: Yes

**********

2. Has the statistical analysis been performed appropriately and rigorously?

Reviewer #1: Yes

Reviewer #2: No

Reviewer #3: Yes

**********

3. Have the authors made all data underlying the findings in their manuscript fully available?

The PLOS Data policy requires authors to make all data underlying the findings described in their manuscript fully available without restriction, with rare exception (please refer to the Data Availability Statement in the manuscript PDF file). The data should be provided as part of the manuscript or its supporting information, or deposited to a public repository. For example, in addition to summary statistics, the data points behind means, medians and variance measures should be available. If there are restrictions on publicly sharing data—e.g. participant privacy or use of data from a third party—those must be specified.

Reviewer #1: Yes

Reviewer #2: No

Reviewer #3: No

**********

4. Is the manuscript presented in an intelligible fashion and written in standard English?

PLOS ONE does not copyedit accepted manuscripts, so the language in submitted articles must be clear, correct, and unambiguous. Any typographical or grammatical errors should be corrected at revision, so please note any specific errors here.

Reviewer #1: Yes

Reviewer #2: No

Reviewer #3: No

**********

5. Review Comments to the Author

Please use the space provided to explain your answers to the questions above. You may also include additional comments for the author, including concerns about dual publication, research ethics, or publication ethics. (Please upload your review as an attachment if it exceeds 20,000 characters)

Reviewer #1: This manuscript has several strengths. It addresses a clinically relevant topic by investigating potential driver genes in psoriasis and their possible therapeutic implications. The integration of multiple scRNA-seq and bulk RNA-seq datasets adds value to the study and may improve the robustness of the analysis. The overall workflow is also reasonably comprehensive, incorporating single-cell analysis, deconvolution, WGCNA, machine learning, cross-dataset validation, pathway analysis, and drug-target prediction. In addition, the study provides a relatively detailed view of cellular heterogeneity in psoriasis and links the identified genes to specific cell populations, particularly basal cells. The use of public datasets and the availability of the analysis code further support the transparency and reproducibility of the work.

However, the following three issues should be noted.

1. The threshold stated in the original text, "each cell needed to contain at least 10 features,"seems somewhat lenient.

2. The criterion "each feature needed to be expressed in at least 300 cells" seems rather stringent for retaining low-abundance yet biologically meaningful genes.

3. The authors classified neutrophils under "lymphoid lineage," while B cells, NK cells, and NKT cells appeared under “myeloid lineage.”They explained this using “The explanation that these populations were ‘sandwiched between’ clusters and that this was hypothesized to result from cell-cell interactions,” but this interpretation seems somewhat subjective.

Reviewer #2: This study integrates multiple public single-cell RNA-seq and bulk RNA-seq datasets to identify key driver genes in psoriasis. Using WGCNA and machine learning (SVM and RFC), the authors identified four genes—DEFB4A, GJB2, SERPINB3, and SERPINB13—as potential therapeutic targets, followed by molecular docking to screen for small molecule compounds. The topic has translational potential and the four identified genes have prior literature support. However, the manuscript suffers from logical gaps, insufficient methodological transparency, overinterpretation of preliminary findings, and language issues that compromise reproducibility and clarity.

Recommendation: Major Revision

Major Issues

1. The role and identification of “basal cells” are not adequately established

The abstract and results emphasize a correlation between hub genes and basal cells, yet basal cells are not introduced in the Background, nor is their functional relevance to psoriasis explained. The Methods do not specify how basal cells were defined or isolated from the scRNA-seq data (e.g., which marker genes were used). Without this foundation, the reader cannot assess why basal cells are central to the analysis or whether the identification was appropriate.

What needs to be addressed: The authors must clarify the biological rationale for focusing on basal cells in the Background, and provide a clear, reproducible definition of how basal cells were identified in the scRNA-seq dataset.

2. The filtering process from WGCNA hub genes to machine learning input is not transparent

The Methods state that 271 hub genes were identified by WGCNA (MM > 0.8, GS > 0.2). The Results then mention that these were intersected with “basal cell differential genes” to obtain 74 genes used for machine learning. This critical intermediate step is missing from the Methods section, making the analysis workflow incomplete and non-reproducible.

What needs to be addressed: The authors must describe in the Methods how the 74-gene set was derived, including the criteria used to define “basal cell differential genes” (e.g., fold-change and significance thresholds). The complete analytical pipeline should be transparent.

3. The selection of the four key driver genes lacks justification

The manuscript states that the top 30 features from each of the four machine learning models were intersected to yield four genes. However, no rationale is provided for choosing “top 30” as the cutoff. Moreover, the importance or weight values for the four genes are not reported, leaving the reader unable to evaluate whether these genes were consistently top-ranked across models or whether the cutoff was arbitrary.

What needs to be addressed: The authors should justify the feature selection threshold (e.g., by showing cumulative importance or stability across cross-validation folds) and provide the specific importance/weight values for the four genes across all models. This will allow assessment of the robustness of the selection.

4. The molecular docking findings are overinterpreted as validated therapeutic strategies

The docking results are presented as if they constitute validated therapeutic strategies, but several compounds have weak clinical evidence (e.g., glutathione, lactic acid) or marginal binding energy (e.g., -1.48 kcal/mol for lactic acid). While cyclosporine is a known psoriasis treatment, its docking with SERPINB3 does not in itself establish a new therapeutic approach. Presenting these preliminary in silico findings as “targeted therapeutic strategies” overstates the conclusions.

What needs to be addressed: The conclusions must be revised to reflect the preliminary nature of the docking results. The authors should clearly differentiate between well-established drugs (e.g., cyclosporine) and speculative candidates, and avoid claiming therapeutic strategies without experimental validation.

5. Multiple testing correction is not addressed

Multiple comparisons are reported throughout (e.g., differential gene expression, pathway enrichment) using nominal P-values without mention of correction for false discovery rate. This raises concerns about inflated type I error rates.

What needs to be addressed: The authors must specify in the Statistical Analysis section whether and how multiple testing was corrected (e.g., Benjamini-Hochberg). If corrections were applied, adjusted P-values should be reported; if not, the authors should explain why and acknowledge this limitation.

Minor Issues

6. Writing quality and formatting consistency

The manuscript contains non-standard terminology, informal phrasing, and inconsistent formatting (e.g., figure citation styles, italicization of P-values). These issues detract from readability and professionalism.

What needs to be addressed: The authors should engage a native English speaker or professional editing service to improve language clarity and scientific tone. Formatting (figure references, statistical notation) should be standardized throughout.

7. Data availability statement requires revision

The statement includes “or available from the corresponding author upon request,” which is not consistent with PLOS’s open data policy. All data used are from public GEO datasets.

What needs to be addressed: The data availability statement should be revised to state that all data are publicly accessible, with accession numbers provided, and remove the “upon request” option.

Reviewer #3: This study brings together multiple cohorts and combines single-cell analysis, deconvolution, WGCNA, machine learning, and functional enrichment to identify psoriasis-associated candidate genes and explore their possible biological relevance. There is clearly a substantial amount of work here, and the overall analytical framework is fairly complete. One of the stronger parts of the paper is the use of WGCNA to connect the cell-related findings with the later gene-prioritization steps, which helps the different parts of the analysis hold together. The study therefore provides some potentially useful information on psoriasis-associated genes. Even so, there are still several concerns about methodological rigor, clarity of presentation, and the extent to which some of the interpretations are supported by the data. For this reason, I think the manuscript would need major revision before further consideration.

1 The scRNA-seq QC thresholds need to be checked more carefully. The requirement that each cell contain at least 10 features is far below. It is usually used in single-cell quality control and would not effectively remove low-quality cells. At the same time, requiring each feature to be expressed in at least 300 cells seems unusually stringent and could remove subpopulation-specific genes or markers from rare cell types. These two thresholds are difficult to understand and may reflect either incorrect parameter reporting or insufficient methodological description. A clearer explanation of the actual settings and the rationale for them would be helpful.

2 The construction of the CIBERSORT reference matrix is not entirely clear. The phrase “Top50 differential genes for each cell subpopulation in psoriasis and healthy samples” can be read in more than one way, and it is hard to tell whether these genes are intended to be cell-type-specific signature genes or genes identified by comparing psoriasis and healthy samples within each cell subpopulation. If the reference was indeed based on within-cell-subpopulation psoriasis-versus-healthy differential genes rather than conventional cell-type-specific signatures, this should probably be acknowledged in the limitations, because such a strategy could mix disease-state signals into the deconvolution and make the estimated cell fractions harder to interpret.

3 In the further subdivision of lymphoid and myeloid cells, several apparently cross-lineage cell types were reported. The manuscript mainly attributes this to lineage-feature mixing caused by cell-cell interactions, but I am not sure the current evidence is sufficient to support such a large interpretive leap. Annotation accuracy, subclustering purity, and the possibility of doublets seem equally important here. At minimum, this issue should be discussed more cautiously, and some uncertainty in these subtype annotations should be acknowledged .

4 The integrated single-cell cohort includes both tissue-derived and peripheral-blood-derived samples.It naturally differ quite substantially in cellular composition. Similar designs do exist in the literature, so this is not inherently unreasonable, but the manuscript does not fully explain whether downstream analyses were stratified by sample source or otherwise controlled for it. Because this kind of heterogeneity could affect single-cell integration, cell-composition comparisons, and later interpretation, it would be better to state this more explicitly, at least in the limitations.

5 There also seems to be an unedited residual sentence in the Methods section on cell annotation, for example “method for cellular annotation of cell clusters.” This should simply be cleaned up.

6 The machine-learning workflow would benefit from a more explicit description. The manuscript mentions train/validation split, grid search, and cross-validation, but it is still not clear whether all of these steps were carried out strictly within the training set. It would be useful to state clearly whether the 74 candidate genes were selected only within the training data, whether grid search and 10-fold cross-validation were restricted to the training set, and whether extraction of the top 30 features was fully isolated from the validation set. If not, that should at least be acknowledged as a limitation.

7 The sentence “extremely strong predictive performance, which is closely related to the importance and weight of the traits” does not read like a standard machine-learning interpretation. The current wording makes the high AUC sound as though it can be directly explained by the “importance and weight of the traits,” which is not very precise. It would read more naturally to say that the selected gene features showed strong discriminatory power.

8 The phrase “gene inlinearity” in the WGCNA section is unusual and likely to confuse readers. Something like “based on gene co-expression patterns” or “based on gene expression correlations” would be much clearer.

9 The pseudotime interpretation for basal cells also feels a little too strong in places. What the current results support more directly is that these key genes show dynamic expression across different differentiation stages. That is not quite the same as showing that they “play a regulatory role” or are specifically related to basal-cell development or senescence. Since this is not the central focus of the paper, a more restrained wording would probably make the manuscript feel more rigorous rather than less.

10 The repeated use of “driver genes” throughout the manuscript may also be stronger than the current evidence allows. In many contexts, “driver” implies a more direct causal or functionally driving role, whereas the present study mainly relies on in silico analyses .So more neutral alternatives such as “key genes,” “candidate genes,” or “psoriasis-associated genes” may be more appropriate unless direct functional validation is added.

11 The latter half of the section on altered pathway activity in Results reads much more strongly than the earlier, more results-oriented parts of the paper. The discussion around Staphylococcus aureus infection, DEFB4A, and estrogen signaling moves rather quickly from pathway-level association to a fairly complete upstream-trigger/downstream-pathogenesis narrative. This part would be more convincing if it were framed more explicitly as hypothesis-generating.

12 The molecular docking analysis is better treated as a supplementary component rather than a strong part of studies of this type. Some of the drug-related interpretations are written too firmly, and the wording in the Discussion and Conclusion around “therapeutic strategies” and “therapeutic promise” would be better toned down.

13 Because of so many analytical modules are integrated in this study, a simple workflow schematic would make the paper much easier to follow. This is important for showing how the analysis moves.

14 The readability of Fig. 1F could be improved. The connection between the bars and the sample labels below is not very clear, which makes the figure a bit hard to follow. It would help to adjust the layout so that each sample label lines up more clearly with its corresponding bar.

15 The current Data Availability Statement may still need some strengthening to better align with the PLOS ONE data policy. Providing the GEO accession numbers and the analysis code is helpful, but if the GitHub repository mainly contains code rather than the processed outputs behind the main figures and statistical analyses, it is still unclear whether all data needed to reproduce the study’s findings are publicly available without restriction. For a study of this type, it would be helpful to make the main source data publicly available as well, including at least the cell-annotation results, sample-level cell proportion tables, CIBERSORT outputs and reference-gene lists, WGCNA module and hub-gene tables, the 74 intersecting genes and top feature lists from the machine-learning analysis, differential gene and enrichment results, AUCell-related outputs, pseudotime-related results, and docking summary tables. In addition, “available from the corresponding author upon request” does not seem fully consistent with the PLOS ONE policy unless specific restrictions are clearly stated. It would therefore be helpful to revise the Data Availability Statement and indicate more clearly where these processed datasets can be accessed.

**********

6. PLOS authors have the option to publish the peer review history of their article (what does this mean?). If published, this will include your full peer review and any attached files.

If you choose “no”, your identity will remain anonymous but your review may still be made public.

**Do you want your identity to be public for this peer review?**  For information about this choice, including consent withdrawal, please see our Privacy Policy.

Reviewer #1: No

Reviewer #2: No

Reviewer #3: **Yes:** shixian cui

**********

[NOTE: If reviewer comments were submitted as an attachment file, they will be attached to this email and accessible via the submission site. Please log into your account, locate the manuscript record, and check for the action link "View Attachments". If this link does not appear, there are no attachment files.]

To ensure your figures meet our technical requirements, please review our figure guidelines: https://journals.plos.org/plosone/s/figures

You may also use PLOS’s free figure tool, NAAS, to help you prepare publication quality figures: https://journals.plos.org/plosone/s/figures#loc-tools-for-figure-preparation.

NAAS will assess whether your figures meet our technical requirements by comparing each figure against our figure specifications.

# Author comment: Author response to Decision Letter 1

**DOI:** 10.1371/journal.pone.0352663.r002

13 May 2026

Reviewer #1: This manuscript has several strengths. It addresses a clinically relevant topic by investigating potential driver genes in psoriasis and their possible therapeutic implications. The integration of multiple scRNA-seq and bulk RNA-seq datasets adds value to the study and may improve the robustness of the analysis. The overall workflow is also reasonably comprehensive, incorporating single-cell analysis, deconvolution, WGCNA, machine learning, cross-dataset validation, pathway analysis, and drug-target prediction. In addition, the study provides a relatively detailed view of cellular heterogeneity in psoriasis and links the identified genes to specific cell populations, particularly basal cells. The use of public datasets and the availability of the analysis code further support the transparency and reproducibility of the work.

However, the following three issues should be noted.

1. The threshold stated in the original text, "each cell needed to contain at least 10 features,"seems somewhat lenient.

Response:

We sincerely thank the reviewer for the careful reading and for pointing out the potential leniency of our QC criteria. Upon re-examining the manuscript and the actual code, we realized that we had inadvertently swapped the descriptions of two key parameters in the earlier version of the Methods section. The actual QC settings we used are as follows:

• min.cells = 10 (genes detected in fewer than 10 cells were removed)

• min.features = 300 (cells with fewer than 300 detected genes were removed)

• percent. mt < 10%

• percent. hb < 1%

These thresholds are in fact comparable to, or more stringent than, the widely used defaults in the scRNA-seq field (e.g., min.cells = 3, min.features = 200, percent. mt < 10-20%). Specifically, our min.features = 300 filter is stricter than the commonly recommended lower bound of 200, and the percent. hb < 1% is a deliberately conservative cutoff adopted to minimize erythrocyte contamination after inspecting the distribution of hemoglobin transcripts in our dataset.

We have now corrected the description in the Methods section of the revised manuscript. We hope this clarification addresses the reviewer’s concern.

2. The criterion "each feature needed to be expressed in at least 300 cells" seems rather stringent for retaining low-abundance yet biologically meaningful genes.

Response:

We sincerely thank the reviewer for this remark, which further prompted us to re-examine our QC description. We realize that this concern actually originates from the same misstatement described in our response to the previous comment (Comment 1). As clarified above, the actual gene-level filtering threshold we applied was min.cells = 10, not 300. The originally worded criterion “expressed in at least 300 cells” was a typographical error that inadvertently swapped the cell-filtering and gene-filtering parameters.

Under the genuine filtering scheme (min. cells = 10), only genes detected in fewer than 10 cells were removed. This is a rather relaxed, widely-adopted default (the Seurat tutorial frequently uses min.cells = 3; a threshold of 5-10 is common for pilot filtering) that is intentionally designed to preserve low-abundance yet biologically meaningful genes. Therefore, we believe our actual parameter does not impose excessive stringency on gene detection.

The corrected description in the Methods section now reads: “Genes detected in fewer than 10 cells were removed.” We hope this clarification resolves the reviewer’s concern.

3.The authors classified neutrophils under "lymphoid lineage," while B cells, NK cells, and NKT cells appeared under “myeloid lineage.”They explained this using “The explanation that these populations were ‘sandwiched between’ clusters and that this was hypothesized to result from cell-cell interactions,” but this interpretation seems somewhat subjective.

Response:

We sincerely thank the reviewer for highlighting this critical point. Upon reflection, we fully agree with the reviewer that attributing this cross-lineage contamination to cell-cell interactions was an over-interpretation and is, as pointed out, unduly subjective.

As the reviewer suggested, we have now reconsidered the origin of these minor heterogeneous clusters. We believe a more probable and conservative explanation stems from our analytical pipeline. In our study, the total single-cell matrix was merged from multiple samples (data sets). Despite batch-effect correction, residual technical noise can persist. In these fine-grained sub-clustering analyses, such noise can occasionally manifest as a small number of one lineage’s cells being pulled into the sub-clustering space of another lineage due to variable capture efficiency or transcriptomic similarity at low-dimensional projections. These are transient analytical artifacts.

Crucially, as described in our workflow, the final step after all sub-clustering was to re-merge and integrate all populations to obtain a unified, definitive cell atlas (total 23 cell types, Fig.1E). This final merging step effectively corrects these artifacts, as cells are re-annotated and collapsed into their true major lineage groups, ensuring that no neutrophils remain mislabeled as lymphoid, nor lymphoid cells as myeloid, in the final results presented to the reader. Our original description failed to adequately communicate this and instead offered an unsupported biological speculation. We have, therefore, comprehensively revised the relevant section in the manuscript to articulate this more cautious, data-processing-centric rationale. We hope the reviewer finds this revised interpretation more appropriate.

Reviewer #2: This study integrates multiple public single-cell RNA-seq and bulk RNA-seq datasets to identify key driver genes in psoriasis. Using WGCNA and machine learning (SVM and RFC), the authors identified four genes—DEFB4A, GJB2, SERPINB3, and SERPINB13—as potential therapeutic targets, followed by molecular docking to screen for small molecule compounds. The topic has translational potential and the four identified genes have prior literature support. However, the manuscript suffers from logical gaps, insufficient methodological transparency, overinterpretation of preliminary findings, and language issues that compromise reproducibility and clarity.

Recommendation: Major Revision

Major Issues

1. The role and identification of “basal cells” are not adequately established

The abstract and results emphasize a correlation between hub genes and basal cells, yet basal cells are not introduced in the Background, nor is their functional relevance to psoriasis explained. The Methods do not specify how basal cells were defined or isolated from the scRNA-seq data (e.g., which marker genes were used). Without this foundation, the reader cannot assess why basal cells are central to the analysis or whether the identification was appropriate.

What needs to be addressed: The authors must clarify the biological rationale for focusing on basal cells in the Background, and provide a clear, reproducible definition of how basal cells were identified in the scRNA-seq dataset.

Response:

We sincerely thank the reviewer for this constructive suggestion. We fully agree that the original manuscript lacked proper contextualization and a reproducible definition of basal cells, which may have obscured the rationale of our study.

• Background revision:

As advised, we have now added a brief statement in the Introduction to establish the biological relevance of basal cells to psoriasis. Specifically, we note that basal cells, as the proliferative keratinocyte population residing in the basal layer of the epidermis, are widely recognized to play a pivotal role in the hyperproliferation and aberrant differentiation characteristic of psoriatic epidermis. This provides the necessary foundation for why this cell population became the focus of our downstream investigation.

• Identification of basal cells in the scRNA-seq data:

We would like to clarify that basal cells were not pre-selected based on a targeted isolation strategy. Instead, they were identified a posteriori from the full, unbiased single-cell transcriptomic landscape. After standard clustering analysis, differentially expressed genes were calculated for each cluster, and the cluster corresponding to basal cells was annotated using a panel of established keratinocyte lineage markers. To ensure full transparency and reproducibility, we have now added a supplementary table (Supplementary Table 1) listing the key markers used to define basal cells. Additionally, the Methods section now explicitly states this annotation pipeline.

We believe these additions adequately address the reviewer’s concerns by providing both the biological context and a clear, reproducible definition of the basal cells that underpin the study’s conclusions.

2. The filtering process from WGCNA hub genes to machine learning input is not transparent

The Methods state that 271 hub genes were identified by WGCNA (MM > 0.8, GS > 0.2). The Results then mention that these were intersected with “basal cell differential genes” to obtain 74 genes used for machine learning. This critical intermediate step is missing from the Methods section, making the analysis workflow incomplete and non-reproducible.

What needs to be addressed: The authors must describe in the Methods how the 74-gene set was derived, including the criteria used to define “basal cell differential genes” (e.g., fold-change and significance thresholds). The complete analytical pipeline should be transparent.

Response:

We thank the reviewer for this valuable suggestion. The omission of the criteria used to define “basal cell differential genes” and the description of how hub genes were intersected with them was indeed a significant oversight in our earlier manuscript. We have now added a complete description of this process in the Methods section. Specifically:

Basal cell differential gene definition: We identified genes differentially expressed between basal cells and all other cell types using the FindMarkers function in Seurat. The criteria were: only positive markers (only.pos = TRUE), expressed in at least 10% of basal cells (min.pct = 0.1), average log2 fold-change > 0.25 (logfc.threshold = 0.25), and adjusted P-value < 0.05.

Intersection: The 271 hub genes were intersected with the basal cell differential gene set, resulting in 74 overlapping genes (Fig. 3A), which were then advanced to the machine learning analysis.

The Methods section has been revised accordingly to include this detailed pipeline, ensuring full transparency and reproducibility.

3. The selection of the four key driver genes lacks justification

The manuscript states that the top 30 features from each of the four machine learning models were intersected to yield four genes. However, no rationale is provided for choosing “top 30” as the cutoff. Moreover, the importance or weight values for the four genes are not reported, leaving the reader unable to evaluate whether these genes were consistently top-ranked across models or whether the cutoff was arbitrary.

What needs to be addressed: The authors should justify the feature selection threshold (e.g., by showing cumulative importance or stability across cross-validation folds) and provide the specific importance/weight values for the four genes across all models. This will allow assessment of the robustness of the selection.

Response:

We sincerely thank the reviewer for this important comment. We fully agree that the choice of “top 30” needs a clear rationale, and that the feature importance values should be transparently reported.

• Justification of the “top 30” feature cutoff

The threshold of top 30 features was selected to strike a balance between capturing the most informative features and reducing noise from low-importance variables. In high-dimensional biological datasets, importance rankings often plateau after the first few dozen features, and retaining a moderate number of features is a pragmatic strategy commonly adopted in similar multi-model feature selection pipelines (e.g., selecting top 10–50 features per model). For our analysis, we tested the stability of the final gene set by varying the cutoff from 20 to 50; the four selected genes (DEFB4A, GJB2, SERPINB3 and SERPINB13) remained stably in the intersection across a wide range, indicating that the selection is not sensitive to the exact cutoff. We have now added this justification to the Methods section.

• Feature importance values of the four key genes

As suggested, we have now provided the specific importance scores (weights) for the four genes across all four machine learning models in Table 1. This pattern confirms that the four genes were not marginal selections driven by an arbitrary cutoff, but rather consistently dominant across models. We believe this transparency fully addresses the reviewer’s concern regarding the robustness of our selection.

4. The molecular docking findings are overinterpreted as validated therapeutic strategies

The docking results are presented as if they constitute validated therapeutic strategies, but several compounds have weak clinical evidence (e.g., glutathione, lactic acid) or marginal binding energy (e.g., -1.48 kcal/mol for lactic acid). While cyclosporine is a known psoriasis treatment, its docking with SERPINB3 does not in itself establish a new therapeutic approach. Presenting these preliminary in silico findings as “targeted therapeutic strategies” overstates the conclusions.

What needs to be addressed: The conclusions must be revised to reflect the preliminary nature of the docking results. The authors should clearly differentiate between well-established drugs (e.g., cyclosporine) and speculative candidates, and avoid claiming therapeutic strategies without experimental validation.

Response:

We sincerely thank the reviewer for this crucial remark. We fully agree that our original presentation overinterpreted the computational docking results by framing them as validated therapeutic strategies. This was an inappropriate conflation of in silico screening with clinical applicability, and we apologize for the overstatement.

In response, we have comprehensively revised the Results and Discussion sections to reflect the strictly preliminary nature of these findings. Specifically:

• We have replaced the phrase “targeted therapeutic strategies” throughout with terms such as “potential candidate compounds” or “preliminary screening of binding compounds,” and made clear that these represent the first step of a discovery pipeline, not clinically actionable strategies.

• We now clearly distinguish between drugs with established clinical evidence in psoriasis (e.g., cyclosporine) and compounds that currently lack such evidence (e.g., glutathione, lactic acid, progesterone). For cyclosporine, we note that its docking with SERPINB3 provides supplementary structural insight but does not itself define a new therapeutic direction; for the other compounds, we explicitly state that their clinical relevance to psoriasis is speculative and requires validation.

• We have added qualifying language in the Discussion, emphasizing that all in silico results require experimental confirmation and that binding energy alone, particularly marginal values such as the -1.48 kcal/mol for lactic acid, is insufficient to propose clinical utility.

• The revised sections now consistently use cautious language (e.g., “screened compounds,” “potential binders,” “warrants further investigation”) and avoid implying that the reported docking data constitute a ready-to-use therapeutic regimen.

We believe these revisions address the reviewer’s concern by accurately calibrating the strength of our claims to the in silico nature of the evidence. The full modified text is provided in the revised manuscript with all changes highlighted.

5. Multiple testing correction is not addressed

Multiple comparisons are reported throughout (e.g., differential gene expression, pathway enrichment) using nominal P-values without mention of correction for false discovery rate. This raises concerns about inflated type I error rates.

What needs to b

Attachment Submitted filename: Response to Reviewers.docx

# Peer review: Decision Letter 1

**DOI:** 10.1371/journal.pone.0352663.r003

**Authors:** Zhengrui Li, Zhengrui Li

14 Jun 2026

<p>Transcriptomic characterization of key psoriasis-associated genes based on single-cell RNA-seq and machine learning

PONE-D-26-09632R1

Dear Dr. Xu,

We’re pleased to inform you that your manuscript has been judged scientifically suitable for publication and will be formally accepted for publication once it meets all outstanding technical requirements.

Within one week, you’ll receive an e-mail detailing the required amendments. When these have been addressed, you’ll receive a formal acceptance letter and your manuscript will be scheduled for publication.

An invoice will be generated when your article is formally accepted. Please note, if your institution has a publishing partnership with PLOS and your article meets the relevant criteria, all or part of your publication costs will be covered. Please make sure your user information is up-to-date by logging into Editorial Manager at Editorial Manager® and clicking the ‘Update My Information' link at the top of the page. For questions related to billing, please contact billing support.

If your institution or institutions have a press office, please notify them about your upcoming paper to help maximize its impact. If they’ll be preparing press materials, please inform our press team as soon as possible -- no later than 48 hours after receiving the formal acceptance. Your manuscript will remain under strict press embargo until 2 pm Eastern Time on the date of publication. For more information, please contact onepress@plos.org.

Kind regards,

Zhengrui Li

Academic Editor

PLOS One

Additional Editor Comments (optional):

Reviewers' comments:

Reviewer's Responses to Questions

**Comments to the Author**

1. If the authors have adequately addressed your comments raised in a previous round of review and you feel that this manuscript is now acceptable for publication, you may indicate that here to bypass the “Comments to the Author” section, enter your conflict of interest statement in the “Confidential to Editor” section, and submit your "Accept" recommendation.

Reviewer #1: All comments have been addressed

Reviewer #3: All comments have been addressed

**********

2. Is the manuscript technically sound, and do the data support the conclusions?

The manuscript must describe a technically sound piece of scientific research with data that supports the conclusions. Experiments must have been conducted rigorously, with appropriate controls, replication, and sample sizes. The conclusions must be drawn appropriately based on the data presented.

Reviewer #1: Yes

Reviewer #3: Yes

**********

3. Has the statistical analysis been performed appropriately and rigorously?

Reviewer #1: Yes

Reviewer #3: Yes

**********

4. Have the authors made all data underlying the findings in their manuscript fully available?

The PLOS Data policy requires authors to make all data underlying the findings described in their manuscript fully available without restriction, with rare exception (please refer to the Data Availability Statement in the manuscript PDF file). The data should be provided as part of the manuscript or its supporting information, or deposited to a public repository. For example, in addition to summary statistics, the data points behind means, medians and variance measures should be available. If there are restrictions on publicly sharing data—e.g. participant privacy or use of data from a third party—those must be specified.

Reviewer #1: Yes

Reviewer #3: Yes

**********

5. Is the manuscript presented in an intelligible fashion and written in standard English?

PLOS ONE does not copyedit accepted manuscripts, so the language in submitted articles must be clear, correct, and unambiguous. Any typographical or grammatical errors should be corrected at revision, so please note any specific errors here.

Reviewer #1: Yes

Reviewer #3: Yes

**********

6. Review Comments to the Author

Please use the space provided to explain your answers to the questions above. You may also include additional comments for the author, including concerns about dual publication, research ethics, or publication ethics. (Please upload your review as an attachment if it exceeds 20,000 characters)

Reviewer #1: The authors have adequately addressed the comments raised in a previous round of review and I feel that this manuscript is now acceptable for publication.

Reviewer #3: The authors have addressed my previous comments satisfactorily. The revised manuscript includes clearer methodological details, more cautious interpretation of the molecular docking results, appropriate discussion of limitations, and improved data availability through the updated GitHub repository with additional processed files.

**********

7. PLOS authors have the option to publish the peer review history of their article (what does this mean?). If published, this will include your full peer review and any attached files.

If you choose “no”, your identity will remain anonymous but your review may still be made public.

**Do you want your identity to be public for this peer review?**  For information about this choice, including consent withdrawal, please see our Privacy Policy.

Reviewer #1: No

Reviewer #3: **Yes:** shixian cui

**********

# Editor report: Acceptance letter

**DOI:** 10.1371/journal.pone.0352663.r004

**Authors:** Zhengrui Li, Zhengrui Li

PONE-D-26-09632R1

PLOS One

Dear Dr. Xu,

I'm pleased to inform you that your manuscript has been deemed suitable for publication in PLOS One. Congratulations! Your manuscript is now being handed over to our production team.

At this stage, our production department will prepare your paper for publication. This includes ensuring the following:

* All references, tables, and figures are properly cited

* All relevant supporting information is included in the manuscript submission,

* There are no issues that prevent the paper from being properly typeset

You will receive further instructions from the production team, including instructions on how to review your proof when it is ready. Please keep in mind that we are working through a large volume of accepted articles, so please give us a few days to review your paper and let you know the next and final steps.

Lastly, if your institution or institutions have a press office, please let them know about your upcoming paper now to help maximize its impact. If they'll be preparing press materials, please inform our press team within the next 48 hours. Your manuscript will remain under strict press embargo until 2 pm Eastern Time on the date of publication. For more information, please contact onepress@plos.org.

You will receive an invoice from PLOS for your publication fee after your manuscript has reached the completed accept phase. If you receive an email requesting payment before acceptance or for any other service, this may be a phishing scheme. Learn how to identify phishing emails and protect your accounts at https://explore.plos.org/phishing.

If we can help with anything else, please email us at customercare@plos.org.

Thank you for submitting your work to PLOS ONE and supporting open access.

Kind regards,

PLOS ONE Editorial Office Staff

on behalf of

Dr. Zhengrui Li

Academic Editor

PLOS One
